# Limiting mitochondrial plasticity by targeting DRP1 induces metabolic reprogramming and reduces breast cancer brain metastases

**DOI:** 10.1038/s43018-023-00563-6

**Published:** 2023-05-29

**Authors:** Pravat Kumar Parida, Mauricio Marquez-Palencia, Suvranil Ghosh, Nitin Khandelwal, Kangsan Kim, Vidhya Nair, Xiao-Zheng Liu, Hieu S. Vu, Lauren G. Zacharias, Paula I. Gonzalez-Ericsson, Melinda E. Sanders, Bret C. Mobley, Jeffrey G. McDonald, Andrew Lemoff, Yan Peng, Cheryl Lewis, Gonçalo Vale, Nils Halberg, Carlos L. Arteaga, Ariella B. Hanker, Ralph J. DeBerardinis, Srinivas Malladi

**Affiliations:** 1 Department of Pathology, University of Texas Southwestern Medical Center, Dallas, TX, USA.; 2 Harold C. Simmons Comprehensive Cancer Center, University of Texas Southwestern Medical Center, Dallas, TX, USA.; 3 Department of Neuroscience, University of Texas Southwestern Medical Center, Dallas, TX, USA.; 4 Department of Biomedicine, University of Bergen, Bergen, Norway.; 5 Children’s Research Institute, University of Texas Southwestern Medical Center, Dallas, TX, USA.; 6 Breast Cancer Research Program, Vanderbilt University Medical Center, Nashville, TN, USA.; 7 Department of Pathology, Microbiology and Immunology, Vanderbilt University Medical Center, Nashville, TN, USA.; 8 Center for Human Nutrition and Department of Molecular Genetics, University of Texas Southwestern Medical Center, Dallas, TX, USA.; 9 Department of Biochemistry, University of Texas Southwestern Medical Center, Dallas, TX, USA.; 10 Department of Internal Medicine, University of Texas Southwestern Medical Center, Dallas, TX, USA.; 11 Howard Hughes Medical Institute, University of Texas Southwestern Medical Center, Dallas, TX, USA.

## Abstract

Disseminated tumor cells with metabolic flexibility to utilize available nutrients in distal organs persist, but the precise mechanisms that facilitate metabolic adaptations remain unclear. Here we show fragmented mitochondrial puncta in latent brain metastatic (Lat) cells enable fatty acid oxidation (FAO) to sustain cellular bioenergetics and maintain redox homeostasis. Depleting the enriched dynamin-related protein 1 (DRP1) and limiting mitochondrial plasticity in Lat cells results in increased lipid droplet accumulation, impaired FAO and attenuated metastasis. Likewise, pharmacological inhibition of DRP1 using a small-molecule brain-permeable inhibitor attenuated metastatic burden in preclinical models. In agreement with these findings, increased phospho-DRP1 expression was observed in metachronous brain metastasis compared with patient-matched primary tumors. Overall, our findings reveal the pivotal role of mitochondrial plasticity in supporting the survival of Lat cells and highlight the therapeutic potential of targeting cellular plasticity programs in combination with tumor-specific alterations to prevent metastatic recurrences.

Metastatic relapses are common in cancer patients considered disease-free after primary diagnosis and treatment^1^. Stem-like disseminated tumor cells with specialized metastatic traits adapt and survive as latent metastases in distal organs by overcoming oxidative stress, nutrient limitation, microenvironmental and immune defenses. Latent metastatic cells that persist as subclinical disease are responsible for late recurrences. Understanding the traits and vulnerabilities of these cells is critical for developing strategies to prevent metastasis^2^.

Metabolic reprogramming is a hallmark of cancer that evolves during metastatic progression^3^. Nutrient availability and dependencies are likely to dictate metabolic reprogramming of disseminated cancer cells. Given that tumor cells in the primary and metastatic sites are metabolically distinct, targeting extracellular nutrient and microenvironmental driven dependencies of disseminated tumor cells is a rational approach to limit progression of metastasis^4–9^. Tumor cells that undergo metabolic reprogramming overcome stress imposed by the microenvironmental and persist or proliferate in distal organs^6,10,11^. However, the precise molecular processes that facilitate metabolic reprogramming in latent metastatic cells to overcome stress and persist after dissemination remain unknown.

Mitochondria, the cellular energy powerhouses, are integral to stress response, as they not only support cellular bioenergetic needs but also generate metabolites that facilitate molecular and epigenomic responses. Mitochondria are dynamic organelles that undergo coordinated cycles of fusion and fission to facilitate adaptations to cellular and extracellular cues^12^. Cancer cells exploit adaptive mitochondrial dynamics to meet energy needs, regulate reactive oxygen species, reprogram cellular metabolism and survive environmental or nutrient stress^13,14^.

In this Article, using mouse models and patient samples, we investigated how disseminated latent metastatic cells in the lipid-rich brain microenvironment meet their cellular energetic demands and persist to initiate overt metastasis. We report mitochondrial plasticity enables fatty acid oxidation (FAO) and survival of latent metastatic cells as subclinical disease. Furthermore, we demonstrate targeting mitochondrial plasticity by attenuating expression of mitochondrial fission regulator dynamin-related protein 1 (DRP1) and through pharmacologic inhibition of DRP1 using a small brain-permeable molecule suppressed growth of latent metastatic cells and reduced brain metastatic burden in preclinical models.

## Results

### Latent cells uptake FAs secreted by reactive astrocytes

Cancer metastases to the central nervous system are lethal. Brain metastatic incidence in breast cancer patients varies with disease subtype and approximately 25–50% of patients with advanced stage breast cancer present with brain metastases^15–17^. Moreover, metachronous brain metastases are common in HER2^+^ (human epidermal growth factor receptor 2) and hormone receptor-positive breast cancer patients that have undergone primary therapy and considered disease-free^17–22^. Current treatment options for brain metastasis are limited and not curative. To understand how disseminated tumor cells persist as subclinical disease and initiate metachronous metastasis, we performed in vivo phenotypic selection in mice and isolated latent brain metastatic (Lat) cells from HCC1954 and SKBR3 HER2^+^ breast cancer cells^6,23–25^.

**Extended Data Fig. 1 |:**
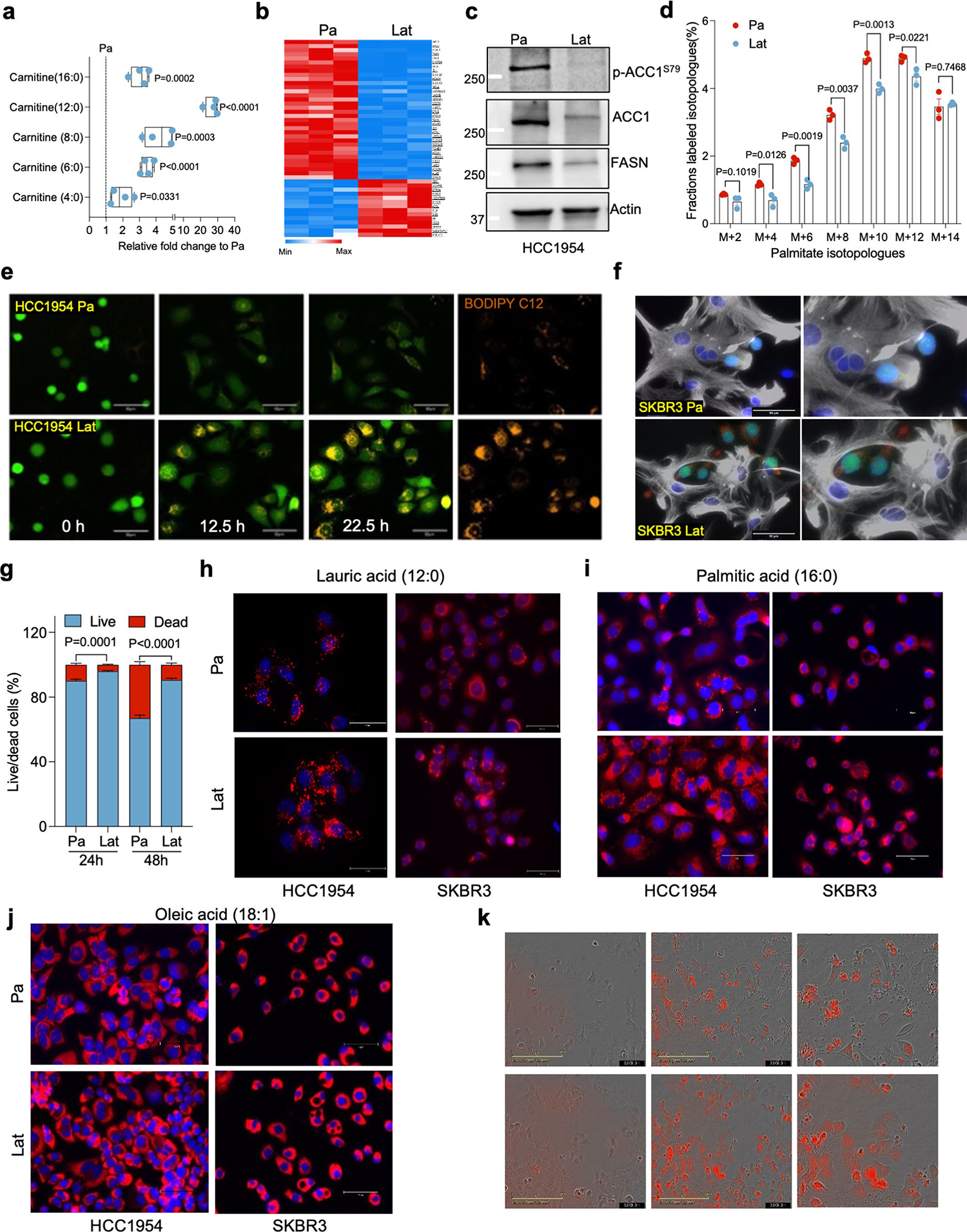
Latent cells uptake fatty acids secreted by reactive astrocytes. **a**. Showing relative difference in steady-state carnitine-conjugated fatty acids between Pa and Lat cells. n = 4, each group. Box and whiskers plot showing minima to maxima with all points. **b**. Heatmap showing differential expression of gene sets related to fatty acid metabolism in HCC1954 Pa and Lat cells. **c**. Western blots showing expression of fatty acid synthesis related enzymes FASN, ACC1 and p-ACC1^S79^ in HCC1954 Pa and Lat cells. **d**. ^13^C_6_-glucose tracing data showing the distribution of ^13^C-labeled even isotopologues of palmitate. n = 3, each group. **e**. Time-lapse confocal images showing BODIPY-558/568-C12 (Orange) transfer from astrocytes to cancer cells (green) in co-culture setting. **f**. Immunofluorescence images showing accumulation of LDs (red) in SKBR3 Pa and Lat cells (green) cultured with BODIPY-558/568-C12 labeled reactive astrocytes (Gray, GFAP staining). **g**. Bar graph showing survival of HCC1954 Pa and Lat cells cultured with astrocytes media. Percentage live/dead cells were quantified at 24 and 48 hours, n = 6, each group. **h-j**. Nile Red staining IF images showing lipid droplets (red) in HCC1954 and SKBR3 Pa and Lat cells treated with lauric acid, palmitic acid, and oleic acid respectively. Briefly, cells were incubated for 24 hours in R3F media and then treated with 100 μM of indicated FAs for 24 hours. Cells were fixed and stained with Nile red (2 μg/ml) for 10 min. **k**. IncuCyte3 time-lapse images (0,24 and 48 hours) showing lipid uptake and cell death in HCC1954 cells cultured with 2 μM of BODIPY-558/568-C12. In **a**, **d**, and **g**, ‘n’ represents biologically independent samples and data presented as mean + /−SEM. P value in **a**, **d**, and **g** were calculated by two-tailed Unpaired t-test. The experiments shown in **c**, **e**, **f**, and **h-k** were repeated independently at least two or three times with similar results.

**Fig. 1 |:**
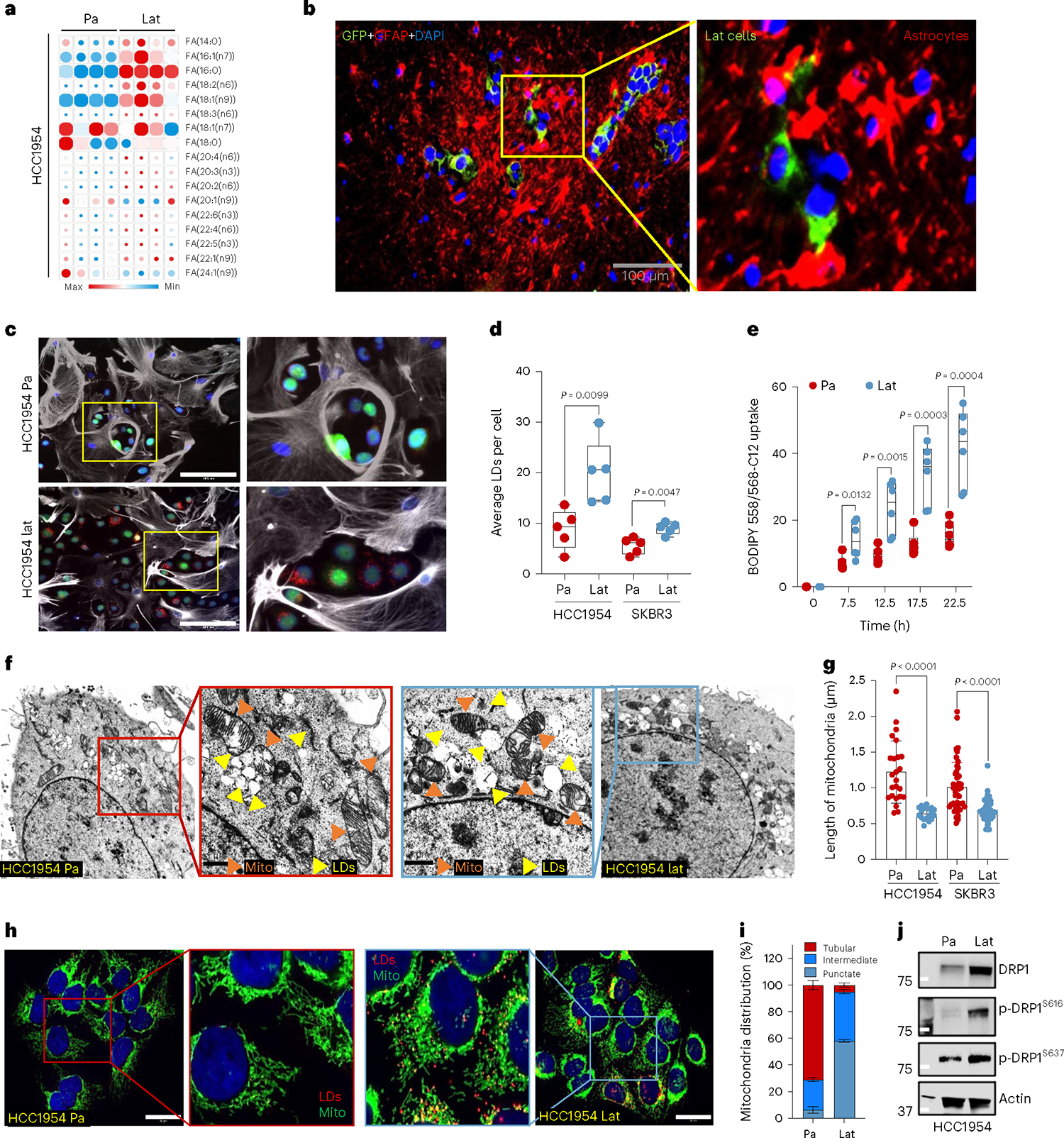
Latent cells uptake FAs secreted by reactive astrocytes. **a**, Heatmap showing total FA profiles of neutral lipid content generated by GC–MS in HCC1954 Pa and Lat cells (*n* = 4, each group). **b**, IF image showing reactive astrocytes (GFAP^+^, magenta) surrounding Lat cells (GFP^+^, green) in mouse brain 5 weeks after intracardiac injection. **c**, IF image showing accumulation of LDs (red) in HCC1954 Pa and Lat cells (green) cultured with BODIPY-558/568-C12 labeled GFAP^+^ reactive astrocytes (gray). Scale bar, 100 μm. **d**, Quantification showing average LDs in Pa and Lat cells (HCC1954 and SKBR3) cultured with BODIPY-558/568-C12 labeled astrocytes for 24 h, *n* = 5 each group. **e**, Quantification showing time-dependent increase in lipid transfer from astrocytes to HCC1954 Pa and Lat cells, *n* = 6, each group. Isolated astrocytes from mice pups were labeled with C12-BODIPY(2 μM) for overnight and then washed three times. Next, HCC1954 Pa and Lat cells cultured in R3F were transferred to the astrocytes cultured plates and live cell imaging was performed using confocal microscope. **d**,**e**, Box and whiskers plot showing minima to maxima. with all points. **f**, TEM images showing LDs and mitochondria in HCC1954 Pa and Lat cells. **g**, Quantification of TEM image showing mitochondrial length in HCC1954 (Pa, *n* = 6; Lat, *n* = 6) and SKBR3 (Pa, *n* = 6; Lat, *n* = 5) model. **h**, Anti-TOMM20 antibody IF images showing mitochondrial morphology of HCC1954 Pa and Lat cells. Scale bar, 50 μm. **i**, Classification of cells based on percentage distribution of tubular, intermediate and punctate mitochondrial morphology in HCC1954 Lat and Pa cells. *n* = 8, each group. **j**, Western blot showing expression of DRP1 along with phosphorylated p-DRP1^S616^(activated form) and p-DRP1^S637^ (inactivated form) in HCC1954 Pa and Lat cells. In **a**, **d**, **e**, **g** and **i**, ‘*n*’ represents biologically independent samples. Data are presented in **d**, **e**, **g** and **i** as mean ± s.e.m., and *P* values in **d**, **e** and **g** were calculated by two-tailed unpaired *t*-test. The experiments shown in **b**, **c**, **f**, **h** and **j** were repeated independently at least two or three times with similar results.

Lat cells are stem cell like, immune evasive and metabolically distinct. Compared to HCC1954 HER2^+^ breast adenocarcinoma parental cells, brain tropic latent metastatic cells were enriched in metabolites and genes associated with fatty acid (FA) metabolism (Extended Data Fig. 1a,b Supplementary Table 1). Correspondingly, significant enrichment in content, namely, carnitine-conjugated FA, lauroyl-l-carnitine (carnitine 12:0) and palmitoyl-l-carnitine (carnitine 16:0), was observed in Lat cells (Extended Data Fig. 1a). Lipidomic profiles also indicated a higher neutral FA content in Lat cells (Fig. 1a and Supplementary Table 2). Cancer cells can synthesize FAs de novo or uptake them from the surrounding microenvironment^26^. However, the expression of FASN, ACC1 and phospho-ACC1 (Ser-79), enzymes associated with FA synthesis, was notably lower in Lat cells (Extended Data Fig. 1c). Moreover, ^13^C_6_-glucose tracing showed significantly low enrichment of palmitate isotopologues (Extended Data Fig. 1d and Supplementary Fig. 1a), suggesting that Lat cells are better equipped to uptake exogenous lipids.

**Extended Data Fig. 2 |:**
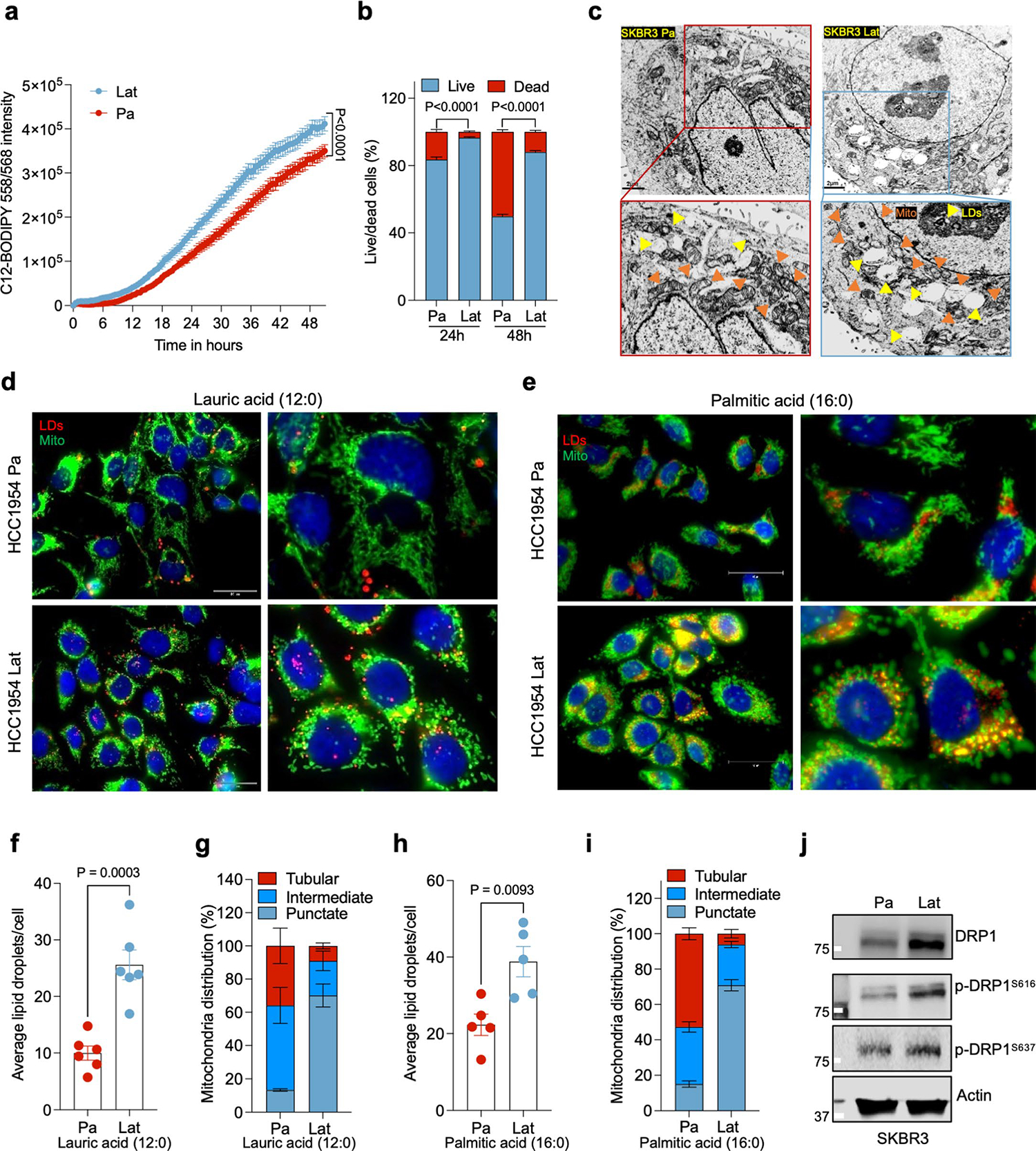
Fragmented mitochondria puncta and LDs enriched in Latent cells. **a**. IncuCyte3 data showing time dependent lipid uptake in HCC1954 Pa and Lat cells in BODIPY-558/568-C12 treated condition. Data were exported as Mean and SEM from n = 25 different fields. P value was calculated by two-tailed Paired t-test. **b**. Bar graph showing survival of HCC1954 Pa and Lat cells cultured with BODIPY-558/568-C12. Percentage live/dead cells were quantified at 24 and 48 hours, n = 6, each group. **c**. Transmission electron microscope (TEM) images showing LDs and mitochondria in SKBR3 Pa and Lat cells. SKBR3 Pa and Lat cells were cultured in MatTek dishes for 24 hours with R3F media followed by treatment of sodium palmitate(100 μM) for 24 hours before processed for TEM. **d** and **e**. IF images of HCC1954 Pa and Lat cells showing LDs (red) and mitochondria (green). Briefly, cells were treated with 100 μM lauric acid or palmitic acid for 24 hours. Cells were fixed and stained with Nile red followed by anti-TOMM20 antibody labeling was performed to visualize LDs and mitochondria respectively. **f**. Bar graph showing LDs, in HCC1954 Pa and Lat cells after lauric acid treatment, n = 6, each group. **g**. Percentage distribution of tubular, intermediate and punctate mitochondrial morphology in HCC1954 Pa and Lat cells treated with lauric acid (n = 4, each group). **h**. Quantification of LDs in palmitate treated HCC1954 Pa and Lat cells. n = 5, each group. **i**. Classification of cells based on percentage distribution of tubular, intermediate and punctate mitochondrial morphology in palmitate treated HCC1954 Pa (n = 5) and Lat (n = 6) cells. **j**. Western blot images showing expression of DRP1 along with p-DRP1^S616^and p-DRP1^S637^ in SKBR3 Pa and Lat cells respectively. In **b** and **f-i**, ‘n’ represents biologically independent samples and data presented as mean + /−SEM. P value in **b, f**, and **h** were calculated by two-tailed Unpaired t-test. The experiments shown in **c, d, e**, and **j** were repeated independently at least two or three times with similar results.

The brain, a lipid-rich organ composed of neurons and supporting glial cells, is a unique microenvironment encountered by disseminated breast cancer cells. Astrocytes, brain resident glial cells, become reactive and undergo molecular and functional remodeling in response to pathologic conditions such as injury, infection or cancer^27^. Indeed, immunofluorescence (IF) staining of mouse brain 5 weeks after intracardiac injection revealed that Lat cells in the brain are surrounded by reactive astrocytes (Fig. 1b). Tumor-associated reactive astrocytes are known to secrete anti-inflammatory cytokines, growth factors, and FAs that promote immune evasion and tumor cell growth^28–30^. Therefore, we established astrocyte cancer cell co-culture assay protocol to assess FA transfer from astrocytes to cancer cells using BODIPY-558/568-C12 (4,4-difluoro-5-(2-thienyl)-4-bora-3a,4a-diaza-s-indacene-3-dodecan oic acid). We first incubated astrocytes with BODIPY-C12 overnight, washed three times with warm culture media and cultured them with GFP^+^ cancer cells. Live cell time-lapse imaging was performed on these co-cultures to assess transfer of fluorescent labeled FAs from astrocytes to cancer cells. Significantly higher number of BODIPY-C12^+^ lipid droplets (LDs) were observed in HCC1954 and SKBR3 Lat cells compared to the Pa cells (Fig. 1c–e, Extended Data Fig. 1e,f, Supplementary Videos 1 and 2 and Supplementary Fig. 1b). Likewise, HCC1954 and SKBR3 exposed to lauric acid (12:0) and palmitic acid (16:0) had increased number of LDs (Extended Data Fig. 1h,i), whereas no such differential effect was observed in the presence of oleic acid (18:1) (Extended Data Fig. 1j). Ability to store excess free FAs from the cytoplasm in the form of LDs attenuates lipo-toxicity and promotes survival^31,32^. Indeed, Lat cells had higher viability compared to parental cells in astrocyte co-culture experiments or upon supplementation of astrocytic media (Extended Data Fig. 1g). Likewise, administration of BODIPY-C12 increased cell death in parental cells compared to Lat cells (Extended Data Figs. 1k and 2a,b and Supplementary Videos 3 and 4). Thus, we conclude Lat cells uptake FAs from astrocytes, store excess as LDs and resist lipo-toxicity induced cell death.

### Fragmented mitochondria puncta enriched in Latent cells

**Extended Data Fig. 3 |:**
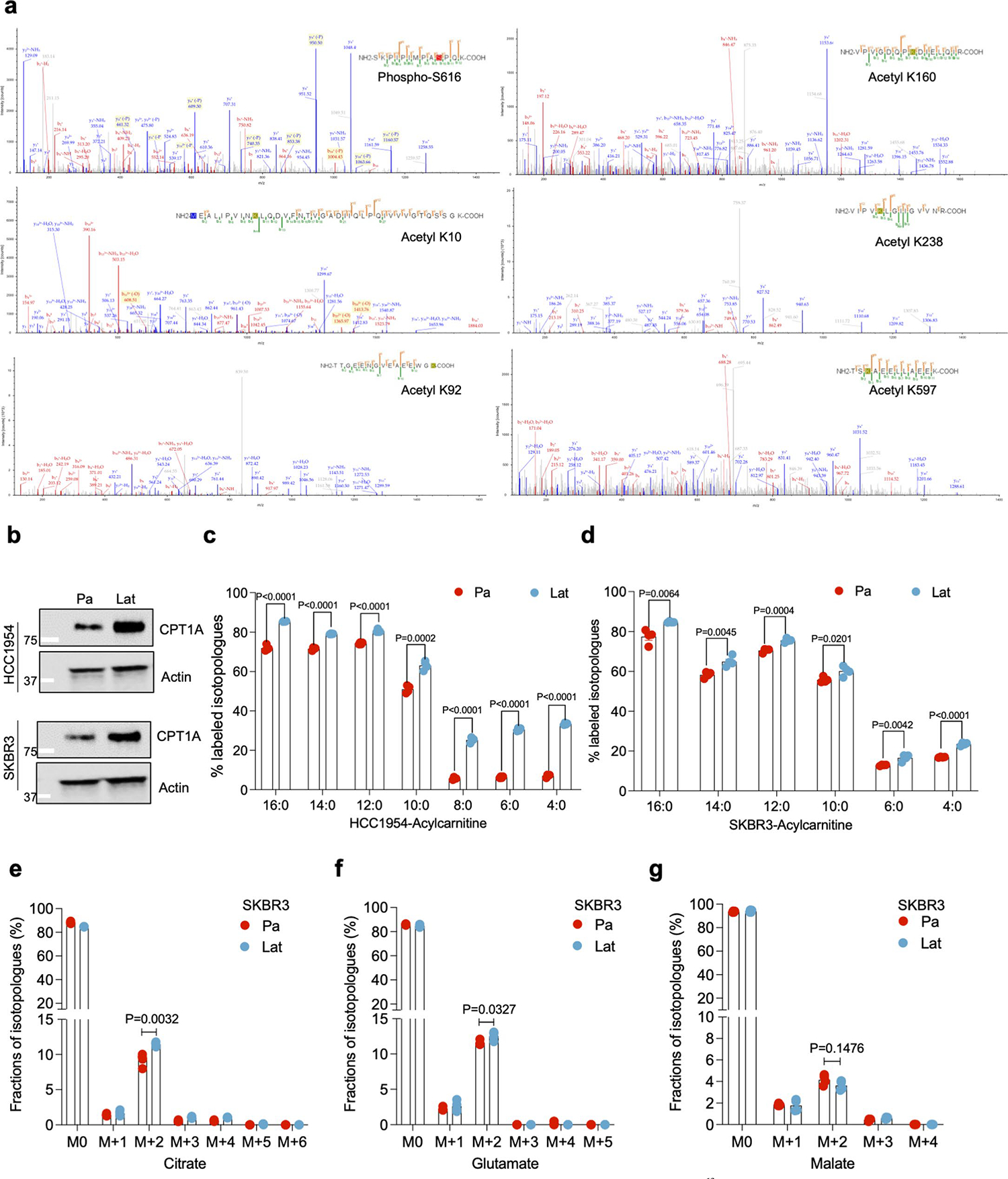
Latent cells oxidize internalized FAs and maintain redox homeostasis. **a**. Mass spectrometry data showing DRP1 post-translational modifications including Serine phosphorylation (S616) and Lysine K10, K92, K160, K238 and K597 acetylation peaks in HCC1954 Lat cells. **b**. Western blots showing expression of carnitine palmitoyl-transferase 1 A (CPT1A) in HCC1954 (upper panel) and SKBR3 (lower panel) Pa and Lat cells. **c** and **d**. ^13^C_16_-Palmitic acid tracing showing enrichment of carnitine-conjugated fatty acids in HCC1954 and SKBR3 Pa and Lat cells. **e-g**. ^13^C_16_-Palmitic acid tracing showing labeled isotopologues of citrate, glutamate, and malate in SKBR3 Pa and Lat cells. In **c-g**, ‘n’ represents biologically independent samples and data presented as mean + /−SEM. P values in **c-g** were calculated by two-tailed Unpaired t-test. The experiments shown in **b** was repeated independently three times with similar results.

To visualize and quantify LDs in Lat and Pa cells we performed transmission electron microscopy. In support of earlier observations, the number of lipid droplets was significantly elevated in Lat cells compared to Pa cells from both cell line models (Fig. 1f and Extended Data Fig. 2c). Noticeably, smaller fragmented mitochondria were observed in Lat cells compared to parental cells that have large tubular mitochondria (Fig. 1f,g and Extended Data Fig. 2c). TOMM20 staining confirmed an increased number of intermediate and punctate mitochondria in Lat cells (Fig. 1h,i). Similarly, increased number of LDs and punctate mitochondria was also observed in HCC1954 Lat cells compared to Pa cells when cultured with lauric acid (12:0) and palmitic acid (16:0) (Extended Data Fig. 2d–i). In agreement with these observations, expression of mitochondrial fission protein, DRP1 and its phosphorylation at serine 616 (p-DRP1^S616^) that promotes DRP1 translocation to mitochondria was elevated in HCC1954 and SKBR3 latent cells (Fig. 1j and Extended Data Fig. 2j)^33,34^. Increased DRP1 acetylation has also been reported to promote DRP1 phosphorylation and mitochondrial fission^35^. Mass spectrometry analysis of DRP1 further confirmed high abundance of p-DRP1^S616^ and identified several acetylated lysine residues (K10, K92, K160, K238 and K597) in Lat cells (Extended Data Fig. 3a).

### Lat cells oxidize internalized FAs and maintain redox homeostasis

**Fig. 2 |:**
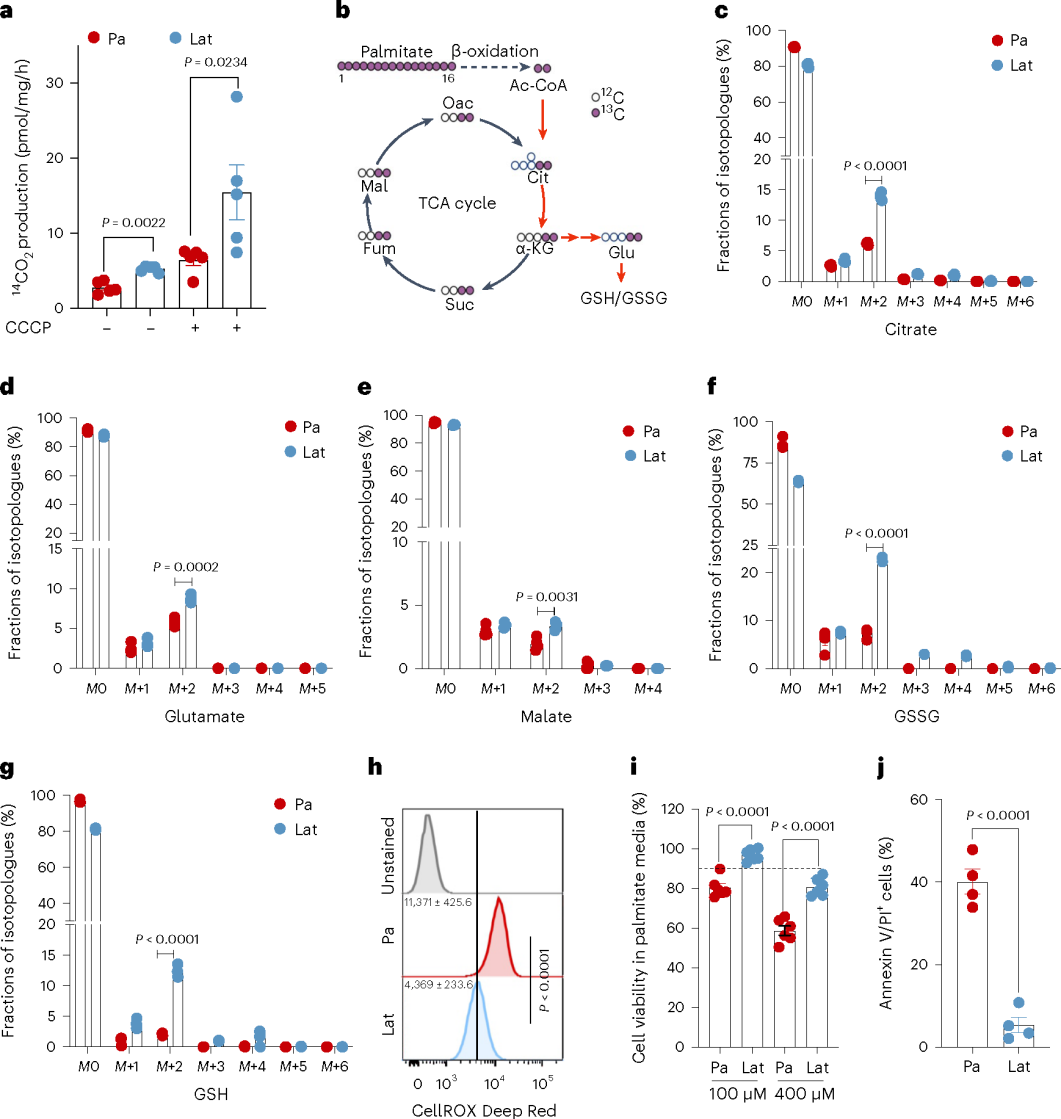
Lat cells oxidize internalized FAs and maintain redox homeostasis. **a**, Bar graph showing oxidation of ^14^C-palmitic acid (1μCi ml^−1^ and production of ^14^CO_2_ in presence of palmitic acid (100 μM), L-carnitine (1 mM) and glucose (10 mM) in Pa and Lat cells. *n* = 5, each group. **b**, Schematic illustration showing distribution of palmitate-derived carbon in TCA cycle intermediates and glutathione. Empty circles represent ^12^C and violet circles represent ^13^C (illustration was made using BioRender.com). **c–g**, ^13^C_16_ palmitic acid tracing showing enrichment of isotopologues of citrate (**c**), glutamate (**d**) and malate (**e**), GSSG (**f**) and GSH (**g**) in HCC1954 Pa and Lat cells, respectively. Briefly, cells were grown in R3F for 24 h and then treated with bovine serum albumin (BSA)-conjugated ^13^C_16_ palmitic acid (100 μM) for 24 h. Samples were collected in 80% methanol in water, and enrichment of metabolites was analyzed by LC–MS. *n* = 4, each group. **h**, Flow cytometry analysis of cellular ROS (CellROX Deep Red staining) in Pa and Lat cells (*n* = 8, each group). **i**, MTT cell viability assay showing survival of HCC1954 Pa and Lat cells (*n* = 6, each group) grown in palmitic acid (100 μM and 400 μM) for 48 h. **j**, Showing APC Annexin V/propidium iodide (PI)^+^ cells in HCC1954 Pa and Lat cells (*n* = 4, each group) treated with palmitic acid for 48 h. In **a** and **c**–**j**, ‘*n*’ represents biologically independent samples. Data are presented in **a** and **c**–**j**, as mean ± s.e.m., and *P* values in **a** and **c**–**j** were calculated by two-tailed unpaired *t*-test.

Internalized FAs could be directed to mitochondria for oxidation or directly stored in LDs. Increased β-oxidation of FAs in mitochondria could generate acetyl-CoA, which feeds into the tricarboxylic acid (TCA) cycle and promotes cell survival during stress or nutrient depleted conditions^36,37^. Indeed, ^14^C-labeled palmitate treatment induced higher ^14^CO_2_ production in Lat cells compared to Pa (Fig. 2a) suggesting increased FAO. Further supporting this observation, carnitine palmitoyl-transferase IA (CPT1A), which converts long-chain fatty acyl-CoA to fatty acylcarnitine and helps translocate FAs into the mitochondria, was enriched in HCC1954 and SKBR3 Lat cells (Extended Data Fig. 3b).

**Extended Data Fig. 4 |:**
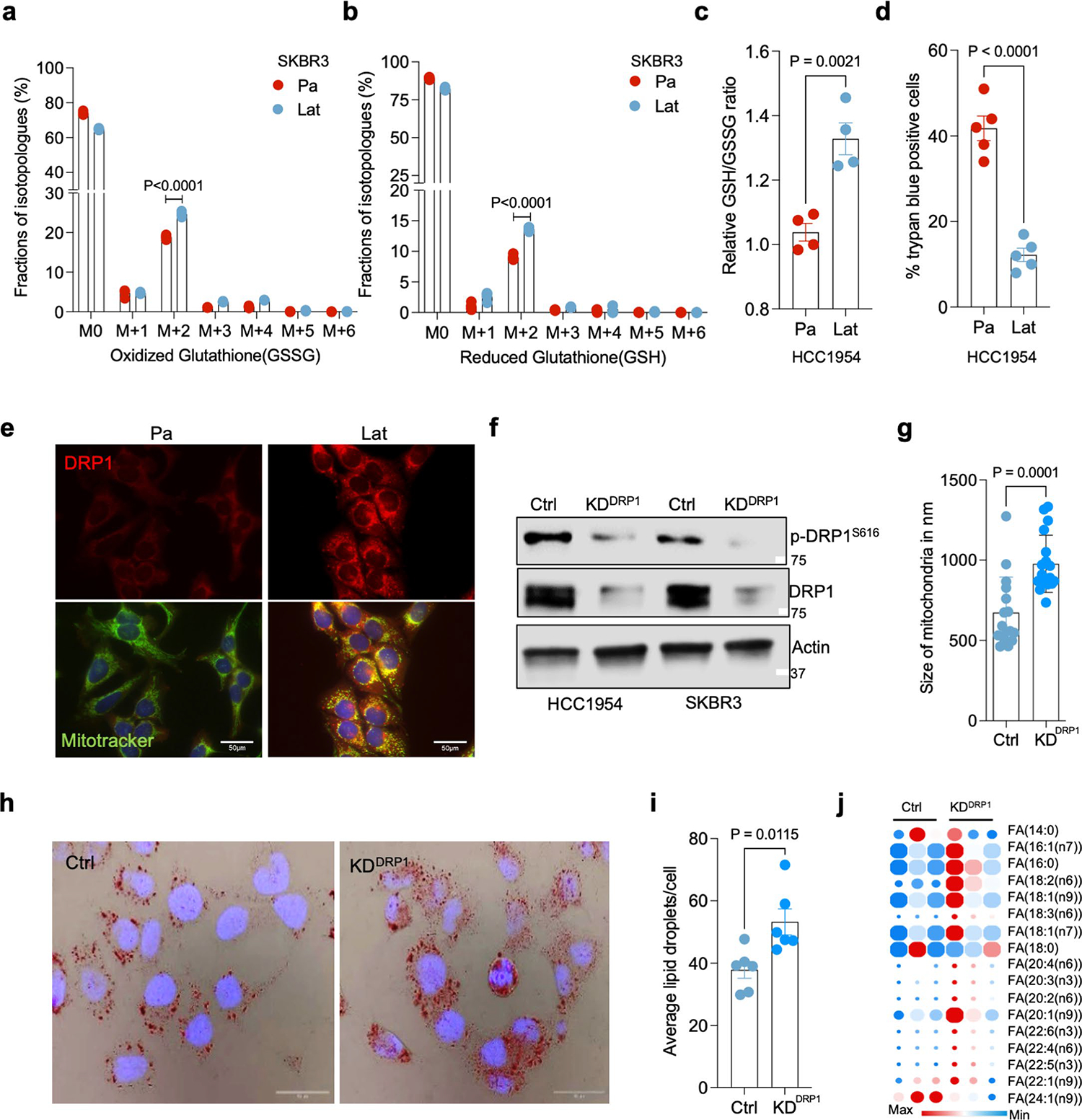
DRP1 promotes mitochondrial fragmentation in Latent cells. **a** and **b**. ^13^C_16_-Palmitic acid tracing showing labeled isotopologues of GSSG and GSH in SKBR3 Pa and Lat cells. **c**. Measurement of steady-state GSH/GSSG ratio between Pa and Lat cells (HCC1954). **a-c**. n = 4, each group. **d**. Trypan blue exclusion assay showing viability of HCC1954 Pa and Lat cells when grown in palmitate (100 μM) for 48 hours. n = 5, each group. **e**. Immunofluorescence images showing localization of DRP1 to mitochondria in HCC1954 Pa and Lat cells. To visualize mitochondria Mitotracker Deep Red FM staining (500 nM, 30 min) was performed. **f**. Western blot images showing DRP1 and p-DRP1^S616^ in DRP1-depleted HCC1954 and SKBR3 Lat cells. **g**. Mitochondrial length quantification in Ctrl and DRP1-depleted HCC1954 Lat cells. n = 3, each group. **h**. Oil red O staining showing differential accumulation of LDs in Ctrl and DRP1-depleted HCC1954 Lat cells. **i**. Quantification of LDs in Ctrl vs DRP1 knockdown HCC1954 Lat cells upon palmitate treatment. n = 6, each group. **j**. Heatmap showing total fatty acid profiles of neutral lipid content generated by GC-MS in Ctrl and DRP1-KD Lat cells. Analysis was performed using 2.5×10^5^ cells in each sample and data were normalized to internal fatty acid standards. n = 3, each group (Fig. 3g and Extended Data Fig. 4j were performed in a single experiment, thus controls are same). In **a-d**, **g, i** and **j**, ‘n’ represents biologically independent samples, data presented as mean + /−SEM and P values were calculated by two-tailed Unpaired t-test. The experiments shown in **e, f** and **h** were repeated independently at least two or three times with similar results.

To further assess FA utilization patterns and their contribution to the observed differences in metabolite pools, we performed ^13^C_16_ palmitate isotope tracing analysis (Fig. 2b). Carnitine-conjugated FAs were significantly high in Lat cells (Extended Data Fig. 3c,d). Furthermore, the M+2 isotopologue of citrate, glutamate, and malate were significantly enriched in HCC1954 and SKBR3 Lat cells compared to Pa cells (Fig. 2c–e and Extended Data Fig. 3e–g). Also, palmitate-derived carbons were enriched in the GSH (reduced glutathione) and GSSG (oxidized glutathione) forms of glutathione in Lat cells (Fig. 2f,g and Extended Data Fig. 4a,b). Steady-state glutathione quantification indicated an elevated GSH/GSSG ratio in Lat cells with reduced cellular ROS (Fig. 2h and Extended Data Fig. 4c). As noted earlier, Lat cells are better equipped to store excess FAs as LDs and survive than parental cells (Fig. 2i,j and Extended Data Fig. 4d). Thus, latent cells oxidize internalized FAs, which then undergo oxidation through the TCA cycle and maintain cellular redox homeostasis that is critical for metastasis initiating cells.

### DRP1-driven mitochondrial dynamics enable FAO and redox homeostasis

**Fig. 3 |:**
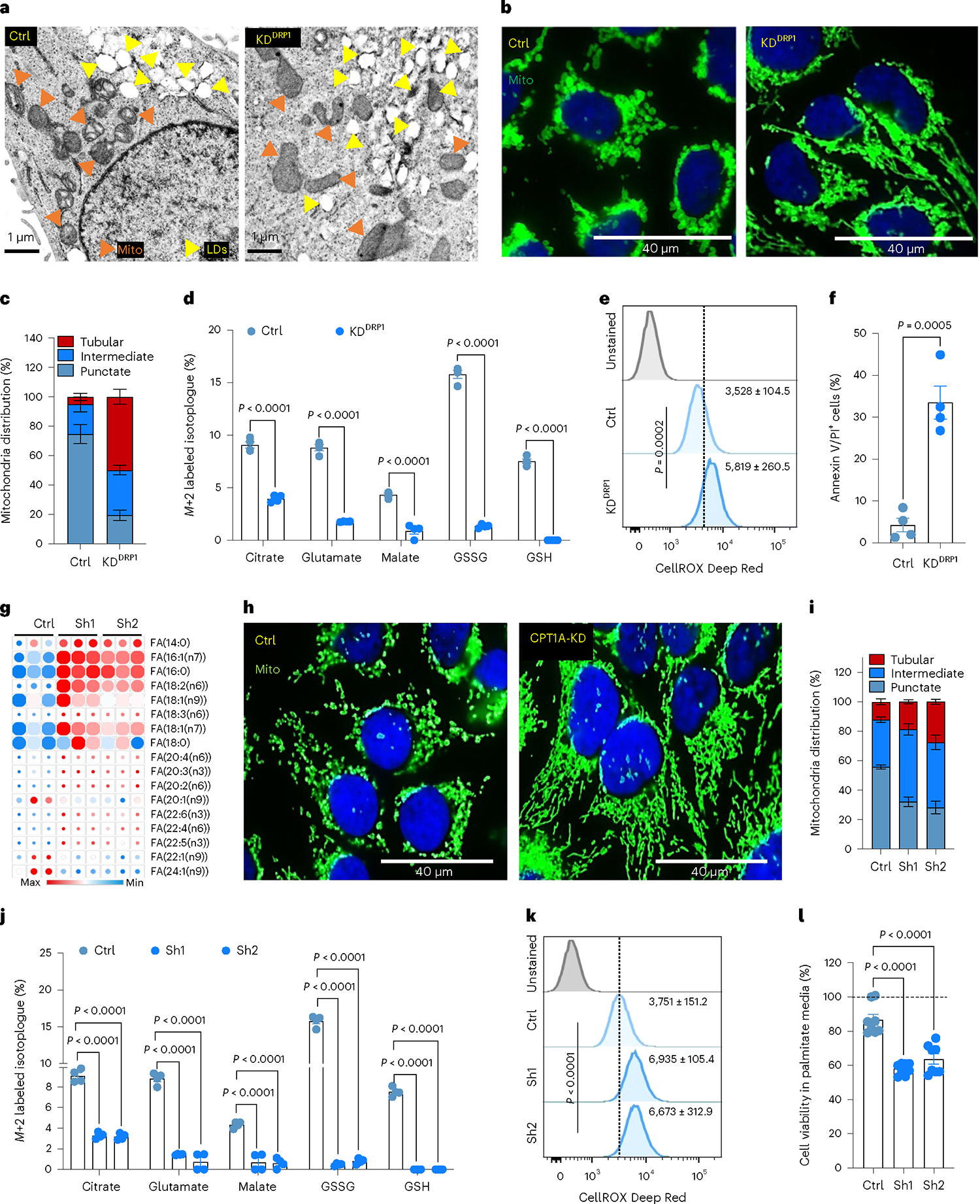
DRP1-driven mitochondrial dynamics enable FAO and redox homeostasis. **a**, TEM images showing LD and mitochondria in Ctrl and DRP1-depleted HCC1954 Lat cells. **b**, IF images highlighting altered mitochondrial dynamics in Ctrl and DRP1-KD Lat cells. **c**, Classification of cells based on percentage distribution of tubular, intermediate and punctate mitochondrial morphology in Ctrl (*n* = 5) and DRP1-KD (*n* = 4) HCC1954 Lat cells. **d**, LC–MS data showing enrichment of M+2 isotopologue of citrate, glutamate, malate, GSSG and GSH from ^13^C_16_-palmitic acid in Ctrl and DRP1-KD HCC1954 Lat cells. *n* = 4, each group. **e**, Flow cytometry analysis of cellular ROS (CellROX Deep Red staining) in Ctrl and DRP1-depleted Lat cells (*n* = 4, each group). **f**. Flow cytometry analysis showing APC Annexin V/PI^+^ cells in HCC1954 Ctrl and DRP1-depleted Lat cells (*n* = 4, each group) treated with 100 μM palmitic acid for 48 h. **g**, Heatmap of neutral lipids in Lat cells showing differential FA content upon CPT1A depletion in HCC1954 Lat cells (*n* = 3). Morpheus, Broad Institute software was used for generating heatmap. **h**, IF images showing mitochondrial morphology in Ctrl and CPT1A knockdown HCC1954 Lat cells. **i**, Quantification of mitochondrial morphology (percentage distribution of tubular, intermediate and punctate mitochondria) in Ctrl and CPT1A-depleted Lat cells, *n* = 5, each group. **j**, Labeling of M+2 isotopologue of citrate, glutamate, malate, GSSG and GSH from ^13^C_16_-palmitate in Ctrl and CPT1A-depleted HCC1954 Lat cells (^13^C_16_-palmitate tracing in DRP1 and CPT1A-depleted cells was performed in a single experimental set up with same control). *n* = 4, each group. **k**, CellROX Deep Red staining showing ROS in Ctrl and CPT1A-depleted HCC1954 Lat cells, *n* = 4, each group. **l**, MTT cell viability assay showing survival of Ctrl and CPT1A-depleted HCC954 Lat cells grown in palmitic acid (100 μM) for 48 h. *n* = 8, each group. In **c**–**g** and **i**–**l**, ‘*n*’ represents biologically independent samples, and data are presented as mean ± s.e.m. *P* values were calculated by two-tailed unpaired *t*-test (**d**–**f**) or ordinary one-way ANOVA (**j**–**l**). The experiments shown in **a**, **b** and **h** were repeated independently at least two or three times with similar results.

As noted earlier, HCC1954 and SKBR3 latent cells had augmented levels of mitochondrial fission protein DRP1 and the activated phosphorylated form p-DRP1^S616^ (Fig. 1j and Extended Data Fig. 2j) that promotes DRP1 translocation to mitochondria and mitochondrial fission (Extended Data Fig. 4e). Moreover, punctate mitochondria are associated with increased FAO^35^. Therefore, we checked to see if altered mitochondrial dynamics facilitated increased FAO and metabolic reprogramming in Lat cells. To test this possibility, we generated DRP1-depleted HCC1954 and SKBR3 Lat cells using doxycycline (dox)-inducible small hairpin RNA (shRNA) (Extended Data Fig. 4f and Supplementary Table 3) targeting *DNM1L* (dynamin 1 like) gene that encodes DRP1 protein. As expected, transmission electron microscope (TEM) and IF analyses show DRP1 depletion in Lat cells results in increased mitochondrial length, tubular mitochondria and reduced number of punctate mitochondria compared to controls (Fig. 3a–c and Extended Data Fig. 4g). LD accumulation was augmented in DRP1-depleted latent cells (Extended Data Fig. 4h,i). In agreement, lipidomic profiles indicated increased FA content in DRP1-depleted latent cells (Extended Data Fig. 4j and Supplementary Table 4).

**Extended Data Fig. 5 |:**
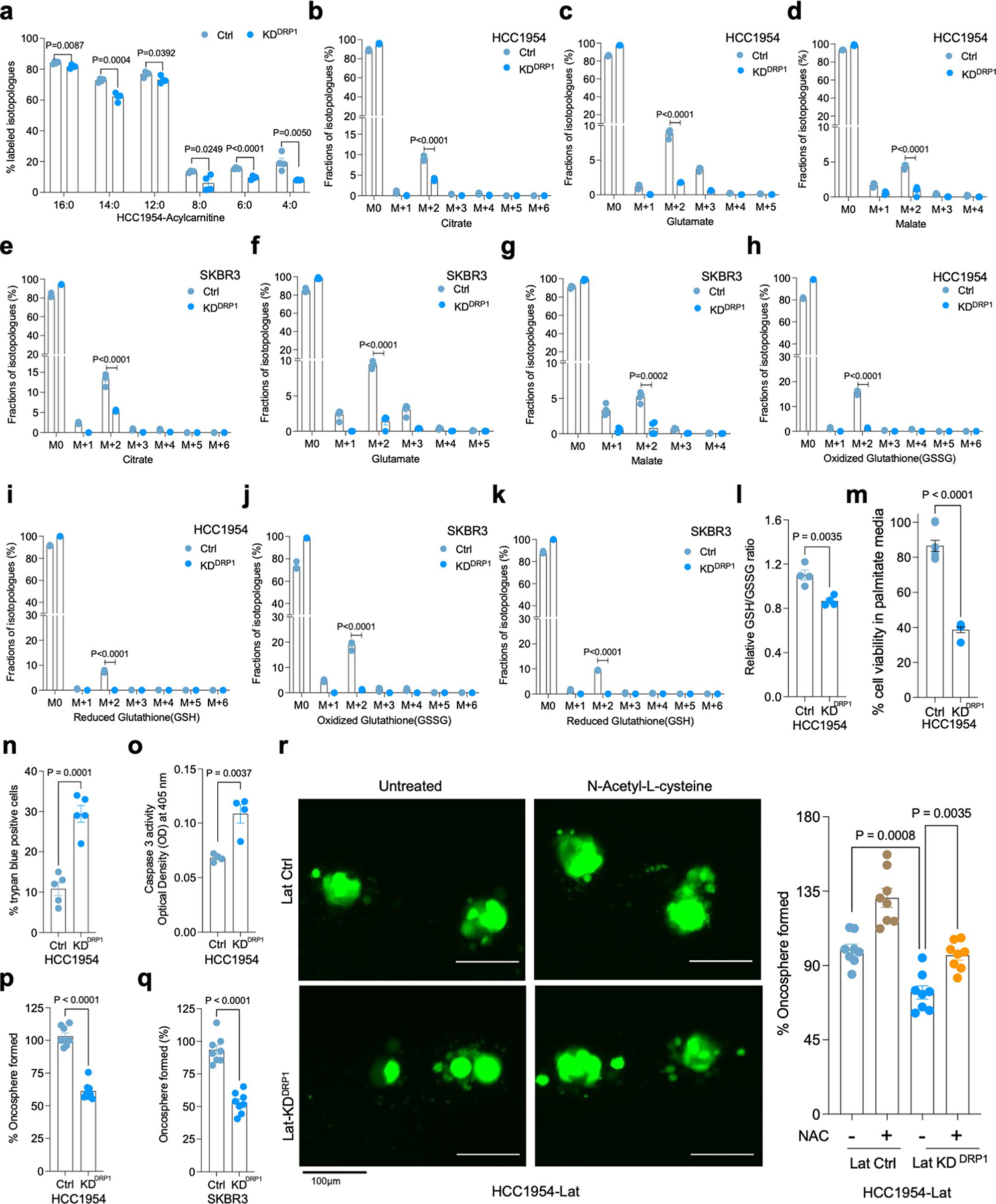
DRP1-driven mitochondrial dynamics enable FAO and redox homeostasis. **a**. ^13^C_16_-Palmitic acid tracing showing enrichment of carnitine-conjugated fatty acids in DRP1-depleted HCC1954 Lat cells. **b-g**. LC-MS data showing enrichment of citrate, glutamate and malate isotopologues from ^13^C_16_-Palmitic acid in HCC1954 and SKBR3 Ctrl and DRP1-KD Lat cells respectively. **h-k**. Labeling of GSH and GSSG from ^13^C_16_-palmitate in HCC1954 and SKBR3 Lat cells (Ctrl and DRP1-KD). **l**. Measurement of relative steady-state GSH/GSSG ratio showing differences between Ctrl and DRP1-KD HCC1954 Lat cells (HCC1954). **a-l**. n = 4, each group. **m**. MTT assay showing cell viability of Ctrl (n = 8) and DRP1-KD (n = 8) HCC1954 Lat cells treated with palmitate(100 μM) for 48 hours (Fig. 3l and Extended Data Fig. 5m was performed in a single experiment with same control). **n**. Trypan blue exclusion assay showing viability of HCC1954 Pa and Lat cells grown in R3F + palmitic acid (100 μM) for 48 hours. n = 5, each group. **o**. Showing caspase-3 activity (Optical density at 405) in Ctrl and DRP1-depleted HCC1954 Lat cells. n = 4, each group. **p** and **q**. Oncosphere formation in Ctrl and DRP1-KD HCC1954 and SKBR3 Lat cells respectively. n = 8, each group. **r**. Showing the effect of treatment of N-acetyl-l-cysteine (NAC; 1 mM) on oncosphere forming ability of Ctrl and DRP1-depleted Lat cells. n = 8, each group. In **a-r**, ‘n’ represents biologically independent samples and data presented as mean + /−SEM. P values in **a-q** were calculated by two-tailed Unpaired t-test and **r**, was calculated by Ordinary one-way ANOVA.

Next, we asked whether altered mitochondrial dynamics impacts FA uptake and oxidation in Lat cells. ^13^C_16_ palmitate tracing in control and DRP1-depleted Lat cells indicated no notable differences in palmitate uptake. However, DRP1 depletion led to reduced synthesis of carnitine-conjugated FAs (Extended Data Fig. 5a) and attenuated fractional enrichment in *M*+2 isotopologue of citrate, glutamate and malate (Fig. 3d and Extended Data Fig. 5b–g). Moreover, DRP1 depletion drastically reduced the levels of glutathione GSH and GSSG (Fig. 3d and Extended Data Fig. 5h–k). In agreement with these observations, DRP1-depleted Lat cells had a significant drop in steady-state glutathione and increased cellular ROS (Fig. 3e and Extended Data Fig. 5l). Moreover, DRP1 depletion results in decreased cell viability due to increased cell death upon palmitate treatment and attenuated formation of oncospheres (Fig. 3f and Extended Data Fig. 5m–q) and administration of antioxidant N-acetyl-l-cysteine (NAC) was able to rescue these observed differences (Extended Data Fig. 5r). These results suggests that DRP1-driven mitochondrial dynamics enable FAO and redox homeostasis in Lat cells.

**Extended Data Fig. 6 |:**
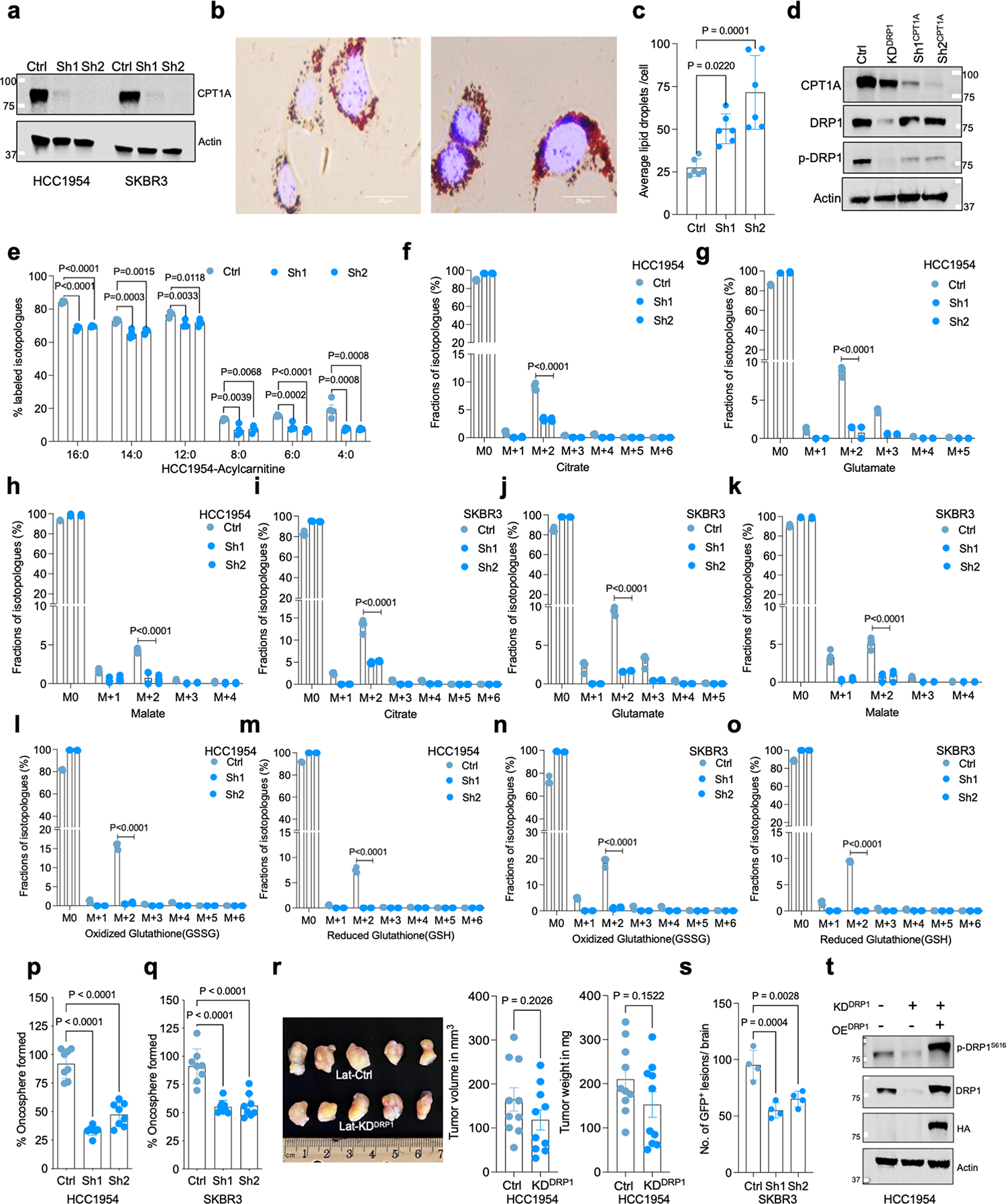
CPT1A aids FAO and altered mitochondrial dynamics in Latent cells. **a**. Western blots showing validation of CPT1A knockdown in HCC1954 and SKBR3 Lat cells. **b**. Oil red O staining images showing differential accumulation of LDs in Ctrl and CPT1A-depleted Lat cells. **c**. Quantification of LDs in Ctrl and CPT1A knockdown Lat cells treated with sodium palmitate (100 μM) for 24 hours, n = 6, each group. **d**. Western blot images showing CPT1A, DRP1 and p-DRP1^S616^ in CPT1A and DRP1-depleted HCC1954 Lat cells. **e**. ^13^C_16_-Palmitic acid tracing showing enrichment of carnitine-conjugated fatty acids in Ctrl and CPT1A-depleted (Sh1, Sh2) HCC1954 Lat cells. **f-k**. LC-MS data showing labeling of citrate, glutamate, and malate isotopologues from ^13^C_16_-Palmitic acid in Ctrl and CPT1A-depleted HCC1954 and SKBR3 Lat cells. (Palmitate tracing in DRP1 and CTP1A depleted cells were performed using same controls). **l-o**. Showing fractions of GSH and GSSG isotopologues labeled from ^13^C_16_-palmitate in Ctrl and CPT1A-depleted HCC1954 and SKBR3 Lat cells. **e-o**. n = 4, each group. **p** and **q**. Showing the ability of oncosphere formation in Ctrl and CPT1A-depleted HCC1954 and SKBR3 Lat cells (n = 8) respectively. **r**. Orthotopic tumor generated from Ctrl (n = 10) and DRP1-depleted (n = 10) Lat cells. Tumors were collected 4 weeks post injection; tumor volume and weight were measured. **s**. Quantification of GFP + brain metastatic lesions in SKBR3 Lat cells (Ctrl and CPT1A-depleted knockdown; n = 4, each group). **t**. Western blot data showing rescue of DRP1 with doxycycline inducible overexpression of HA-tagged full-length DRP1 in DRP1-depleted cells Lat cells. In **c**, and **e-q**, ‘n’ represents biologically independent samples. **r** and **s** ‘n’ represent number of tumor and number of mice respectively. **c**, and **e-s**, data presented as mean + /−SEM. P values in **c, e-q** and **s** were calculated by Ordinary one-way ANOVA and **r**, two-tailed Unpaired t-test was used. The experiments shown in **a, b, d**, and **t** were repeated independently at least two or three times with similar results.

As we observed, DRP1-driven mitochondrial plasticity promotes FAO and helps survival of Lat cells. We checked the effect of enriched CPT1A expression in Lat cells by knocking down CPT1A with dox-inducible shRNA (Extended Data Fig. 6a and Supplementary Table 3). Consequences of limiting CPT1 function are increased accumulation of LDs and lipotoxicity^38,39^. Indeed, oil red staining showed an increase in number of LDs (Extended Data Fig. 6b,c). Moreover, FA profiles also indicated an increase in FA content upon CPT1A depletion (Fig. 3g and Supplementary Table 4).

Noticeably distinct changes in mitochondrial dynamics, a notable reduction in punctate mitochondria and increased mitochondrial length or tubular mitochondria, were observed upon CPT1A depletion (Fig. 3h,i). Moreover, p-DRP1^S616^ levels were attenuated upon CPT1A depletion (Extended Data Fig. 6d). ^13^C_16_ palmitate tracing showed no substantial differences in palmitate uptake between Ctrl and CPT1A-depleted cells. However, synthesis of carnitine-conjugated FAs and labeling of M+2 isotopologue of citrate, glutamate, and malate was significantly attenuated upon CPT1A depletion in Lat cells (Fig. 3j and Extended Data Fig. 6e–k). CPT1-depleted HCC1954 and SKBR3 Lat cells had a drastic reduction in glutathione (GSH and GSSG) levels (Fig. 3j and Extended Data Fig. 6l–o). Further phenocopying DRP1 depletion, reduced ability to form oncospheres, increased cellular ROS levels, and increased cell death upon palmitate administration were observed in CPT1-depleted Lat cells (Fig. 3k,l and Extended Data Fig. 6p,q).

### DRP1-driven mitochondrial plasticity promotes metastatic latency

**Fig. 4 |:**
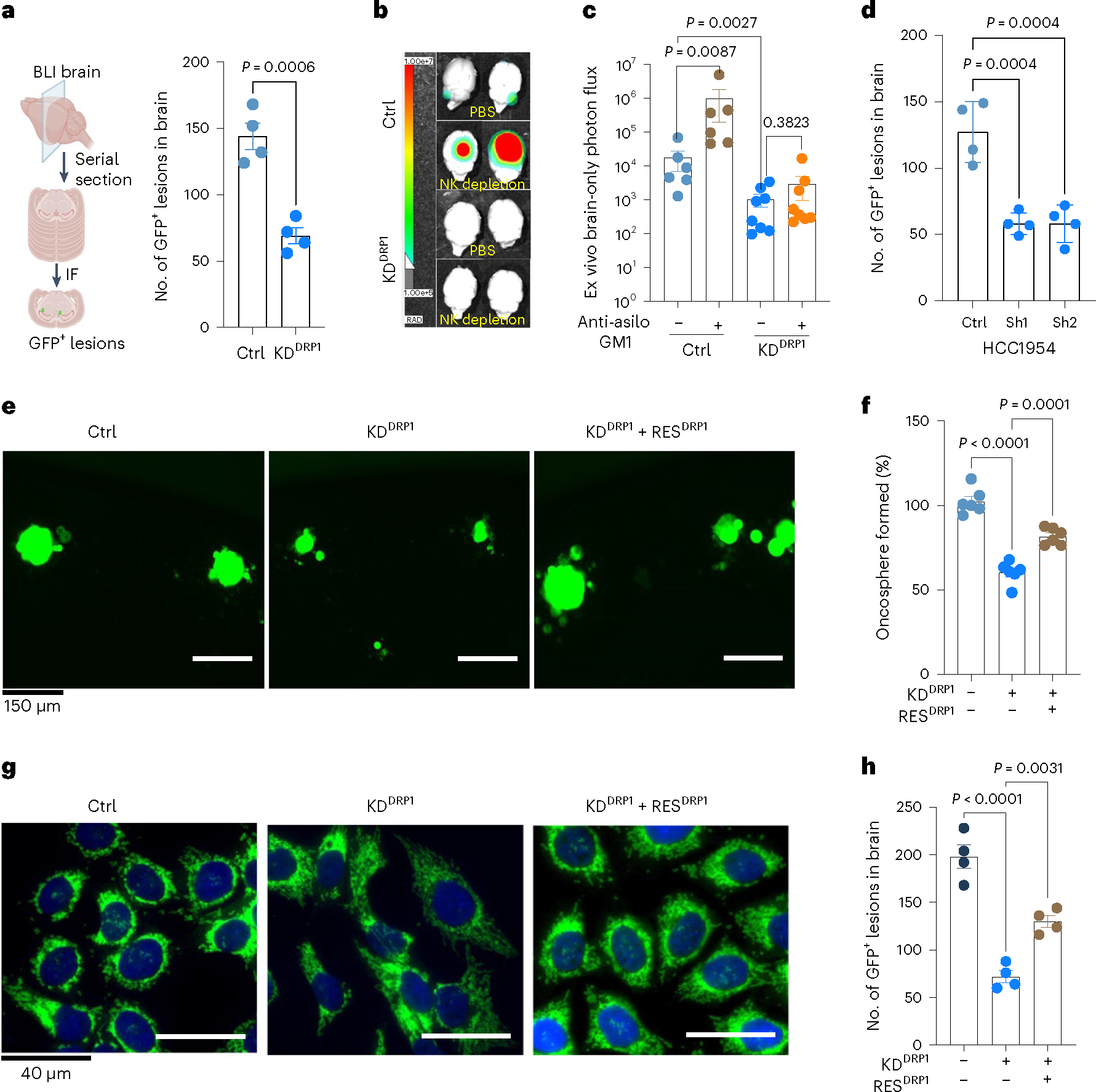
DRP1-driven mitochondrial plasticity promotes metastatic latency. **a**, Brain serial section and quantification of GFP^+^ brain tropic latent metastatic cells/lesions in Ctrl and DRP1-depleted HCC1954 Lat cells, *n* = 4, each group. Illustration was made using BioRender.com. BLI, bioluminescence imaging. **b**,**c**, Ex vivo brain images and bioluminescence signal showing brain-only photon flux in Ctrl (*n* = 6), Ctrl+ anti-asialo-GM1(*n* = 6), KD^DRP1^ (n = 8), and KD^DRP1^ + anti asialo-GM1 (*n* = 8). Here, mice were injected with Ctrl and DRP1-depleted Lat cells, followed by vehicle or anti asialo-GM1 treatment. **d**, Brain serial section and quantification of GFP^+^ brain tropic latent metastatic cells/lesions in Ctrl and CPT1A knockdown HCC1954 Lat cells, *n* = 4, each group. **e**,**f**, Showing oncosphere image and quantification in Ctrl, DRP1-depleted and DRP1-rescued HCC1954 Lat cells. *n* = 6, each group. **g**, IF images showing mitochondrial morphology in Ctrl, DRP1-depleted and DRP1-rescued HCC1954 Lat cells. **h**, Brain serial section and quantification of GFP^+^ brain tropic latent metastatic cells/lesions in HCC1954 Ctrl, DRP1-depleted and DRP1-rescued Lat cells, *n* = 4, each group. In **a**, **c**, **d**, and **h**, ‘*n*’ represents the number of mice, and in **f** represents biologically independent samples, with data presented as mean ± s.e.m. *P* values were calculated by two-tailed unpaired *t*-test (**a**), two-tailed Mann–Whitney *U*-test (**c**) or ordinary one-way ANOVA (**d**, **f**, **h**). The experiment shown in **g** was repeated independently two times with similar results.

To assess the effect of DRP1 depletion on metastatic latency, we intracardially injected Ctrl and DRP1 knockdown Lat cells into mice. One week after injection, mice were supplemented with a dox diet to deplete DRP1. Five weeks after injection, we euthanized mice and collected brains and performed immunohistochemical (IHC) analysis to assess the number of surviving latent cells. Compared to controls, mice bearing DRP1-depleted HCC1954 Lat cells had a significant reduction in the number of GFP^+^ metastatic lesions (Fig. 4a). In contrast, no significant difference in primary tumor (PT) burden (tumor weight and tumor volume) was observed upon DRP1 depletion (Extended Data Fig. 6r). Previously we reported natural killer (NK) cells can limit the proliferation of Lat cells and enforce metastatic latency. Depletion of NK cells with anti-asialo-GM1 polyclonal antibody after dissemination in athymic mice bearing Lat cells results increased metastatic outbreaks^23^. No overt metastasis was observed upon NK cell depletion in mice injected with DRP1-depleted Lat cells (Fig. 4b,c), indicating elimination of residual disease.

As CPT1A loss phenocopies DRP1 depletion, we assessed the effect of CPT1A depletion on metastatic latency by injecting mice intracardially with either Ctrl or CPT1A-depleted HCC1954 and SKBR3 Lat cells. CPT1 depletion was induced by administrating dox diet a week after injection. Analogous to DRP1 depletion, CPT1A depletion resulted in a significant reduction in the number of GFP^+^ metastatic events in mice brains (Fig. 4d and Extended Data Fig. 6s).

Next, we performed DRP1 rescue experiments to show DRP1 is essential for survival of Lat cells. For these studies, we generated inducible HA-tagged *DNM1L* full-length constructs and transduced their expression in DRP1-depleted Lat cells (Extended Data Fig. 6t and Supplementary Table 5). Inducible DRP1 expression resulted in reversing the mitochondrial morphology and augmented the oncosphere-forming ability in DRP1-depleted cells (Fig. 4e–g). As expected, increased number of surviving Lat cells were observed in the brain upon DRP1 rescue (Fig. 4h). Together, our data demonstrate that DRP1-dependent mitochondrial plasticity facilitates FAO and promotes survival of latent metastatic cell in mice.

### Phospho-DRP1 is elevated in human metachronous brain metastases

**Extended Data Fig. 7 |:**
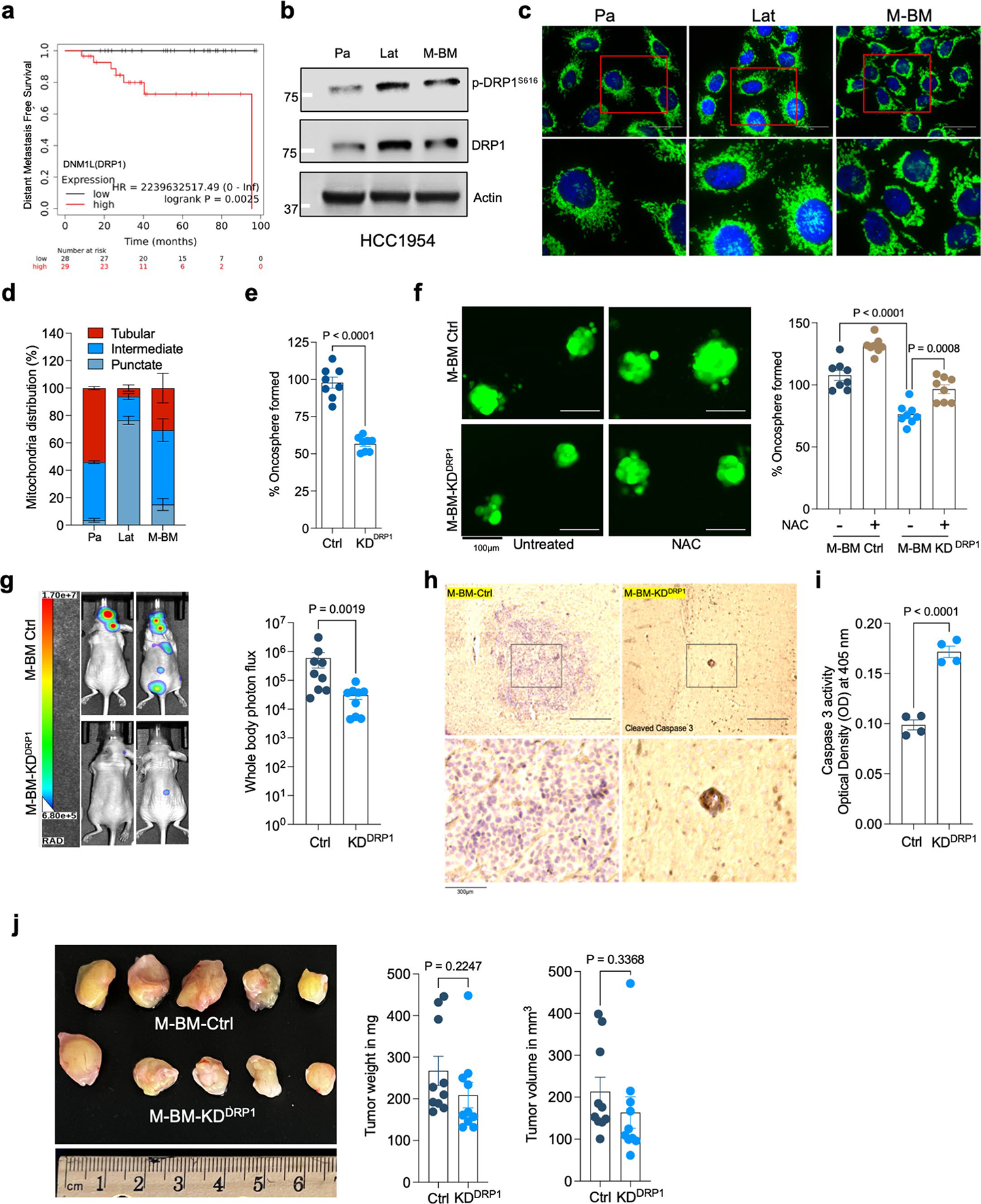
DRP1 is essential for metachronous brain metastases. **a.** Representative Kaplan-Meier plotter showing distant metastasis free survival for breast cancer patients with high or low expression of *DNM1L* (DRP1) gene. **b.** Western blot images showing expression of DRP1 and p-DRP1^S616^ in HCC1954 Pa, Lat and M-BM cells. **c**. Anti-TOMM20 antibody immunofluorescence images showing mitochondrial morphology of HCC1954 Pa, Lat, and M-BM cells. **d**. Classification of cells based on percentage distribution of tubular, intermediate and punctate mitochondrial morphology in HCC1954 Pa, Lat, and M-BM cells (n = 4, each group). **e**. Oncosphere formation in Ctrl and DRP1-KD HCC1954 M-BM cells. n = 8, each group. **f**. Showing the effect of treatment of N-acetyl-l-cysteine (NAC; 1 mM) on oncosphere forming ability of DRP1-depleted HCC1954 M-BM cells (n = 8, each group). **g**. Mice image with *whole body* photon flux showing metastatic burden in mice bearing HCC1954 Ctrl (n = 9) and DRP1(n = 9) depleted M-BM cells. **h**. Immunohistochemical (IHC) staining for cleaved caspase-3 (1:150, DAB, 10X) in Ctrl and DRP1-depleted M-BM cells injected mice brain section. **i**. Graph showing caspase-3 activity in Ctrl and DRP1-depleted HCC1954 M-BM cells. n = 4, each group. **j**. Orthotopic tumor generated from Ctrl and DRP1-depleted M-BM cells (n = 10, each group), post 4 weeks of cell injection. In **d-f**, and **i**, ‘n’ represents biologically independent samples, however **g**, and **j** ‘n’ represents number of mice and number of tumors respectively. In **d-g, I**, and **j** data presented as mean + /−SEM. P values in **e**, and **j** were calculated by two-tailed Unpaired t-test, **f** and **g** were calculated using Ordinary one-way ANOVA and two-tailed Mann–Whitney test respectively. The experiments shown in **b, c**, and **h** were repeated independently at least two to three times with similar results.

**Extended Data Fig. 8 |:**
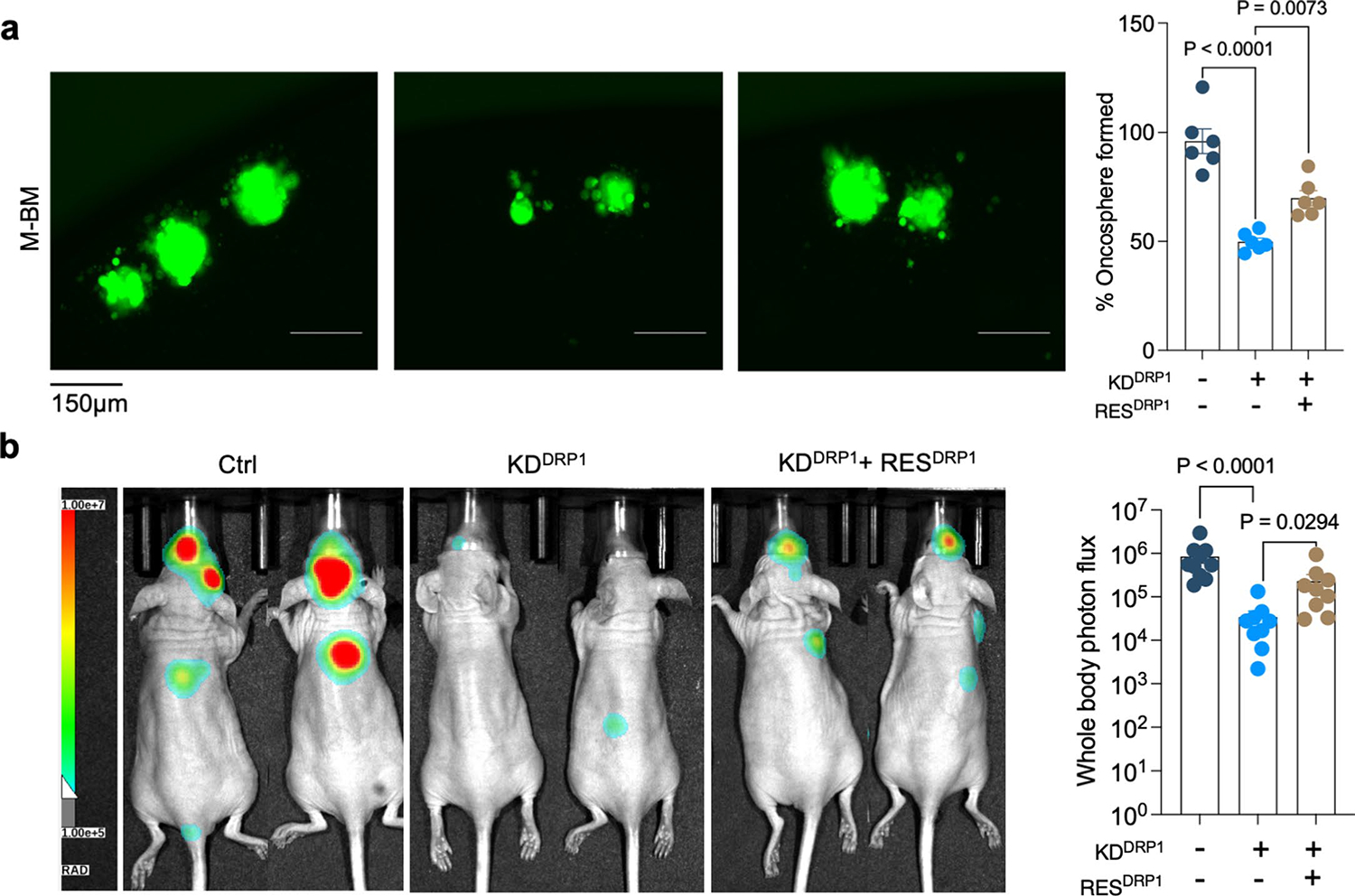
Ectopic expression of DRP1 rescues DRP1-depleted phenotype. **a**. Showing oncosphere image and quantification in Ctrl, DRP1 knockdown and DRP1-rescued HCC1954 Lat cells (n = 6, each group). Data presented as mean + /−SEM. P values were calculated using Ordinary one-way ANOVA. **b**. *Whole body* image and photon flux showing metastatic burden in mice bearing Ctrl (n = 10), DRP1-depleted (n = 9) and DRP1-rescued (n = 9) M-BM cells. Data presented as mean + /−SEM. P value was calculated by Kruskal–Wallis test. In **a** ‘n’ represents biologically independent samples and **b** ‘n’ represents number of mice.

**Fig. 5 |:**
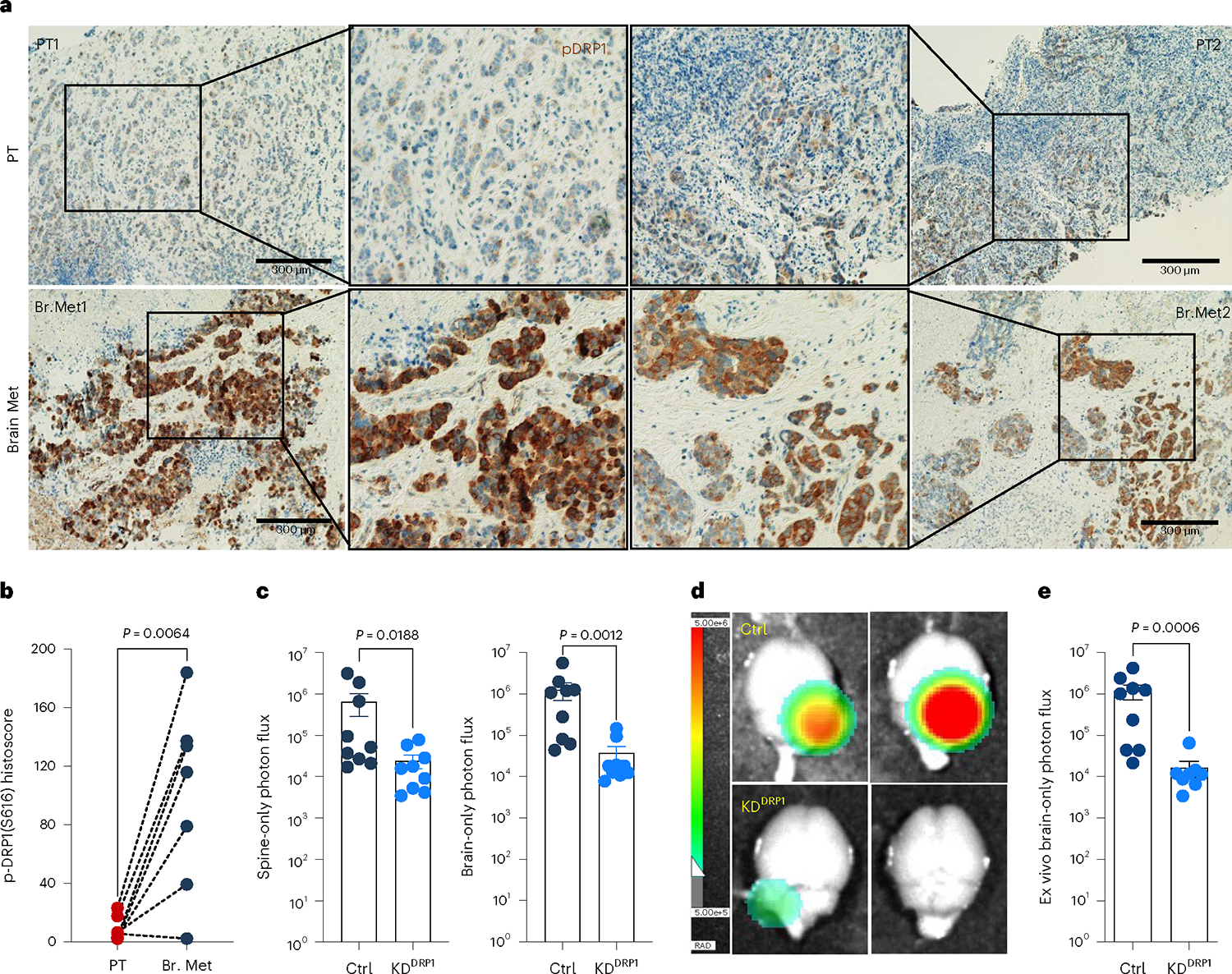
Phospho-DRP1 is elevated in human metachronous brain metastases. **a**, IHC staining for p-DRP1^S616^ (1:200, DAB, 10×) in matched human HER2^+^ PT and metachronous brain metastases (Br. Met). **b**, Representative graph showing histoscore of p-DRP1^S616^ in HER2^+^ PT and matched brain metastatic samples (*n* = 7, each group). **c**, Bar graph showing spine and brain-only photon flux in mice bearing Ctrl (*n* = 9) and DRP1-depleted (*n* = 9) M-BM cells. **d**,**e**, Ex vivo brain images (**d**) and brain-only photon flux (**e**) showing metastatic burden in mice bearing Ctrl (*n* = 9) and DRP1-depleted (*n* = 8) M-BM cells. In **b**, ‘*n*’ represents number of human patients, and in **c** and **d**, ‘*n*’ represents number of mice. Data are presented as mean ± s.e.m. *P* value in **b** was calculated by two-tailed paired *t*-test, and in **c** and **d**, *P* values were calculated by two-tailed Mann–Whitney *U*-test.

**Fig. 6 |:**
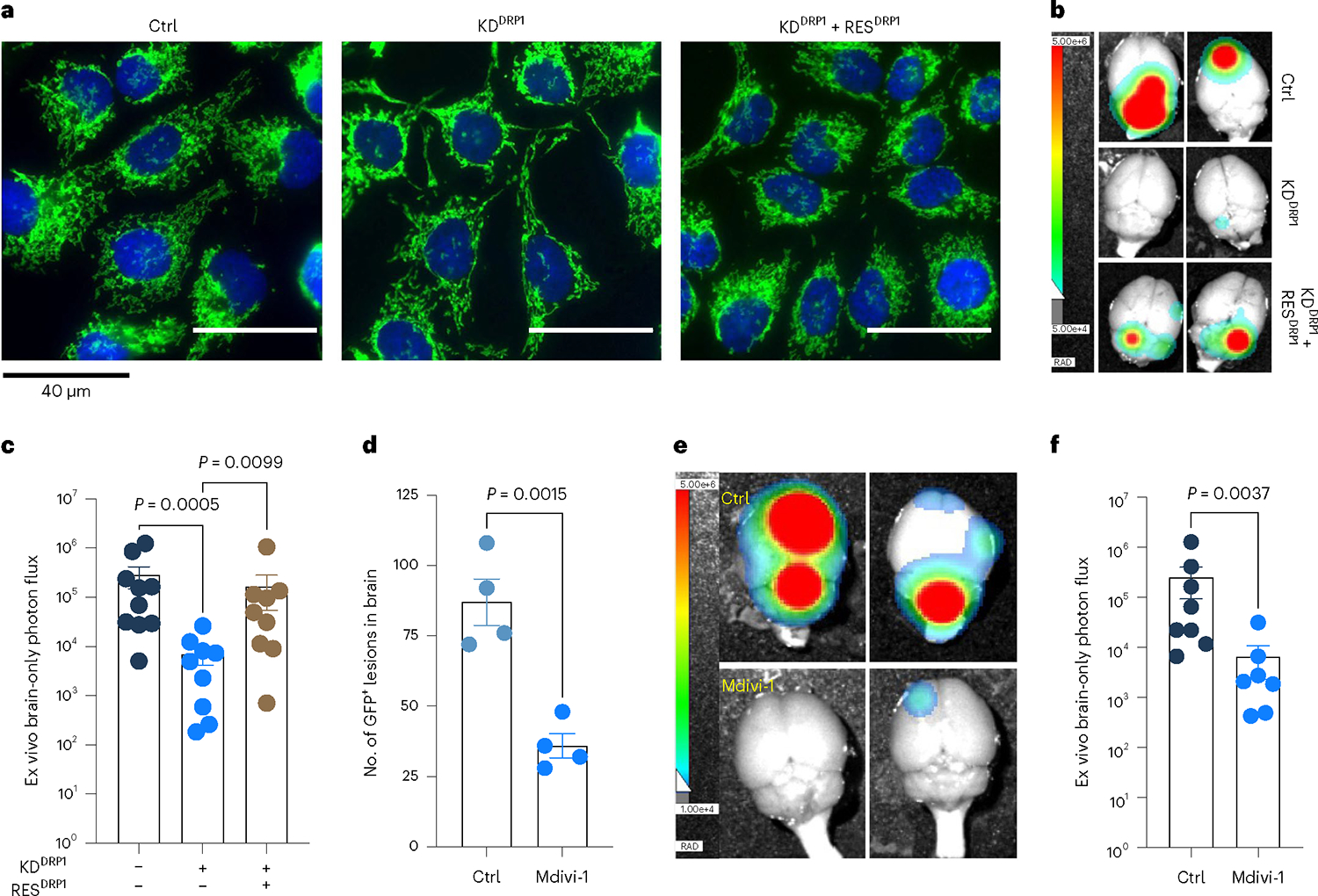
Genetic depletion or pharmacologic inhibition of DRP1 attenuates brain metastasis. **a**, IF images showing mitochondrial morphology of HCC1954 Ctrl, DRP1-depleted and DRP1-rescued M-BM cells. **b**,**c**, Ex vivo brain images (**b**) and brain-only photon flux (**c**) showing metastatic burden in mice bearing Ctrl (*n* = 10), DRP1-depleted (*n* = 9) and DRP1-rescued (*n* = 9) M-BM cells. **d**, Quantification of GFP^+^ brain tropic latent metastatic cells/lesions in mice bearing HCC1954 Lat cells treated with vehicle (10% DMSO in corn oil) and Mdivi-1 (40 mg kg^−1^) for 4 weeks (once daily). *n* = 4, each group. **e**,**f**, Ex vivo brain images (**e**) and brain-only photon flux (**f**) showing metastatic burden in mice bearing M-BM cells. After injection of M-BM cells, mice were treated with either vehicle (10% DMSO in corn oil, *n* = 8), or Mdivi-1 (40 mg kg^−1^, *n* = 7) for 4 weeks (once daily). In **c**, **d** and **f**, ‘*n*’ represents number of mice, and data are presented as mean ± s.e.m. *P* values in **c**, **d** and **f** were calculated by Kruskal–Wallis test, two-tailed unpaired *t*-test and two-tailed Mann–Whitney *U*-test, respectively. The experiment shown in **a** was repeated independently two times with similar results.

High DRP1 expression correlated with poor distant metastasis free survival in HER2^+^ breast cancer patients (Extended Data Fig. 7a). Obtaining biopsies from patients who show no detectable disease after their primary diagnosis is impractical. Therefore, we investigated p-DRP1 status in metachronous brain metastatic lesions from HER2^+^ breast cancer patients^6^, who had undergone a considerable latent metastatic phase (Supplementary Table 6). IHC analysis of matched PT and metachronous brain metastatic lesions from seven HER2^+^ breast cancer patients revealed high expression of p-DRP1^S616^ in brain metastatic lesions compared to corresponding matched PTs (Fig. 5a,b). Likewise, increased DRP1 and p-DRP1^S616^ expression and altered mitochondrial dynamics were observed in metachronous brain metastatic (M-BM) cells compared to Pa and Lat cells (Extended Data Fig. 7b–d)^6^. Akin to Lat cells, depletion of DRP1 in M-BM cells resulted in reduced oncosphere formation which was rescued by administration of antioxidant NAC (Extended Data Fig. 7e,f). M-BM cells predominantly metastasize to the brain with occasional metastasis to the spine/bone. Whole body, spine and brain photon flux analysis indicated a significant decrease in brain and spine/bone metastatic burden upon DRP1 depletion, resulting from increased apoptosis (Fig. 5c–e and Extended Data Fig. 7g–i). Similar to Lat cells, no significant difference in tumor burden (tumor volume and tumor weight) was observed between orthotopically injected Ctrl and DRP1-depleted M-BM cells (Extended Data Fig. 7j). Furthermore, ectopic expression of DRP1 in DRP1-depleted M-BM cells was able to rescue mitochondrial dynamics, oncosphere-forming ability and metastatic incidence in mice (Fig. 6a–c and Extended Data Fig. 8a,b).

### Pharmacologic inhibition of DRP1 attenuates brain metastasis

**Extended Data Fig. 9 |:**
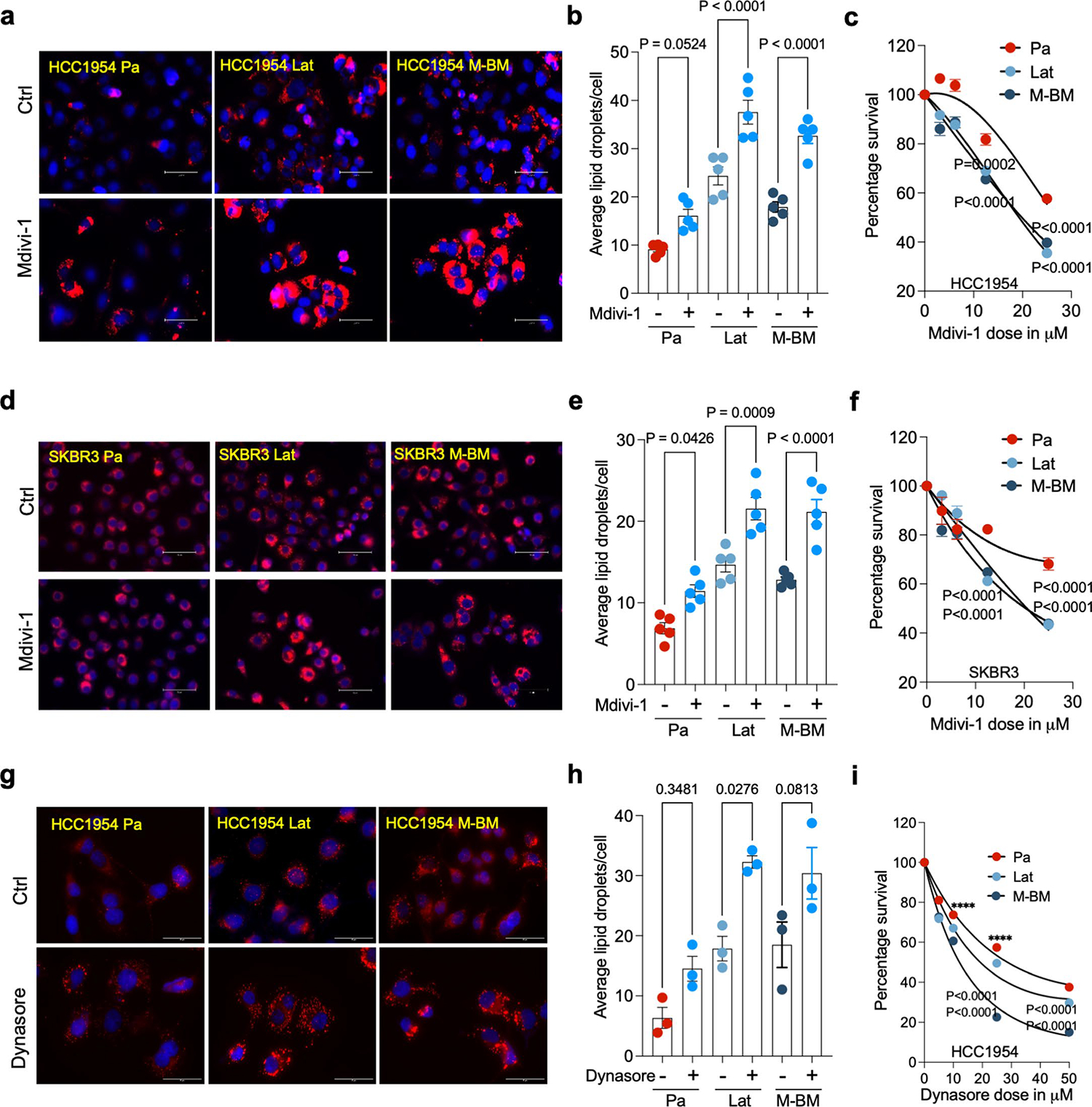
DRP1 inhibitors increases LDs and decreases survival of Latent and M-BM cells. **a**. IF images of HCC1954 Pa, Lat, and M-BM cells with or without Mdivi-1 treatment (12.5 μM, 48 hours). **b**. Quantification of number of LDs between Ctrl and Mdivi-1 treated HCC1954 Pa, Lat, and M-BM (n = 5, each group) cells. **c**. Showing viability of HCC1954 Pa, Lat, and M-BM (n = 8, each group) cells upon Mdivi-1 treatment (0, 3.125, 6.25,12.5 and 25 μM) for 48 hours. **d**. IF images of SKBR3 Pa, Lat, and M-BM cells with or without Mdivi-1 treatment (12.5 μM, 48 hours). **e**. Quantification of number of LDs in SKBR3 Pa, Lat, and M-BM (n = 5, each group) cells after Mdivi-1 treatment. **f**. Showing viability of HCC1954 Pa, Lat, and M-BM (n = 8, each group) cells upon Mdivi-1 treatment (0, 3.125, 6.25,12.5 and 25 μM) for 48 hours. **g**. IF images of Ctrl and Dynasore treated HCC1954 Pa, Lat, and M-BM cells. **h**. Quantification of number of LDs in Ctrl and Dynasore treated (25 μM) HCC1954 Pa, Lat, and M-BM (n = 3, each group) cells. **i**. Showing viability of HCC1954 Pa, Lat, and M-BM (n = 8, each group) cells upon treatment of Dynasore (0, 5, 10,25 and 50 μM) for 48 hours. In **b, c, e, f, h** and **i**, ‘n’ represents biologically independent samples and data presented as mean + /−SEM and P values were calculated by Ordinary one-way ANOVA. The experiments shown in **a**, **d**, and **g** were repeated independently two times with similar results.

Mitochondrial division inhibitor 1 (Mdivi-1) is reported to inhibit DRP1-dependent mitochondrial fission and attenuate neuronal apoptosis in several models of brain ischemia and neurodegeneration^40–42^. As Mdivi-1 is brain-permeable and not neurotoxic, we assessed its effect on Lat and M-BM cells. Similar to DRP1 depletion, Mdivi-1 treatment resulted in increased LDs and reduced viability of HCC1954 and SKBR3 Lat and M-BM cells (Extended Data Fig. 9a–f). Treatment with Dynasore, which interferes with the GTPase activity of the mitochondrial dynamin DRP1 and dynamin 1 and dynamin 2, but not of other small GTPases, resulted in increased LD and reduced cell viability in Lat and M-BM cells (Extended Data Fig. 9g–i)^43^.

**Extended Data Fig. 10 |:**
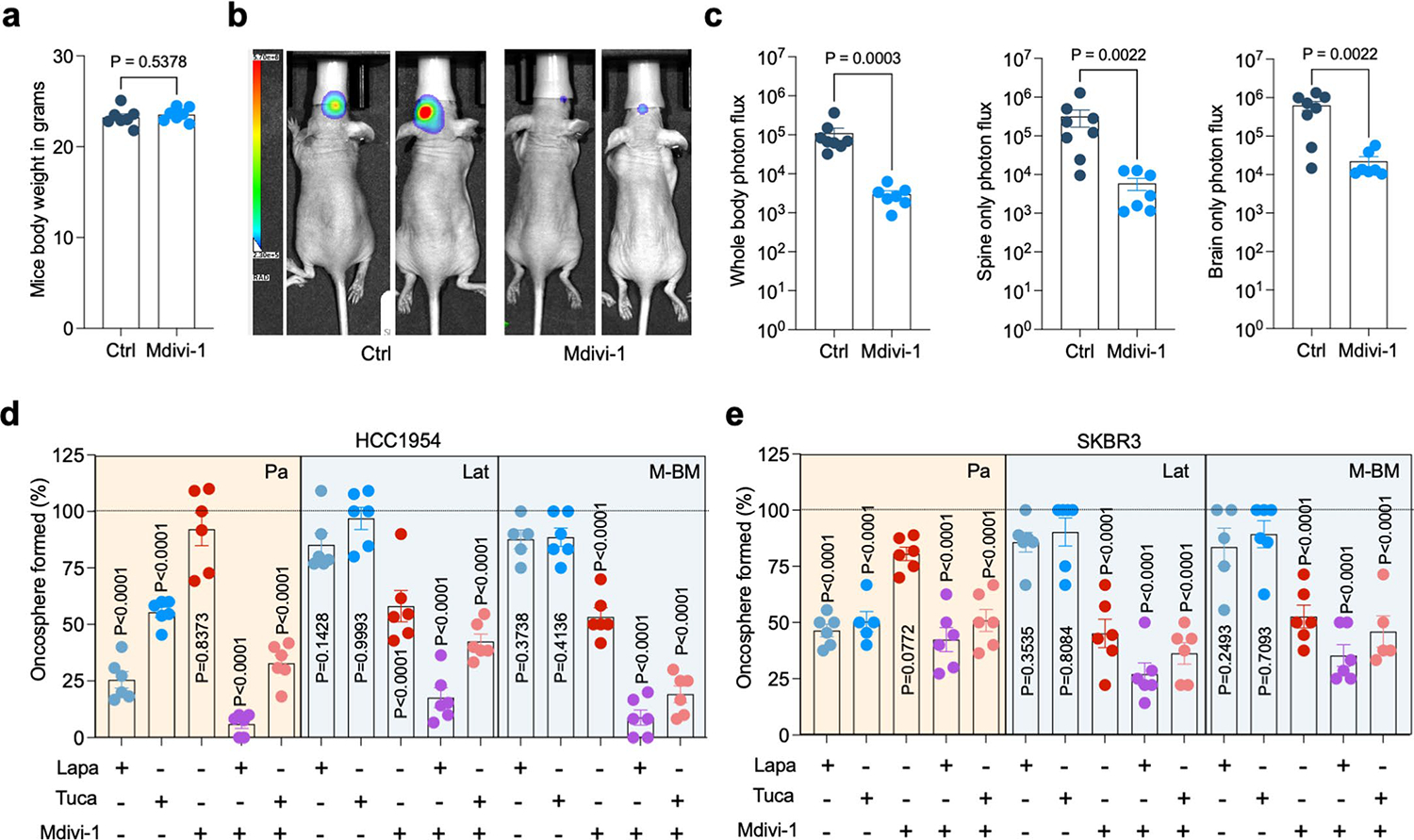
Pharmacologic inhibition of DRP1 attenuates brain metastasis. **a**. Mice body weight comparison after treatment with vehicle (10% DMSO in corn oil, n = 7) and Mdivi-1(40 mg/kg, n = 7) for 4 weeks. Data presented as mean + /−SEM, Unpaired t-test. **b** and **c**. Mice image with *whole body, spine and brain-only* photon flux showing metastatic burden in mice bearing HCC1954 M-BM cells treated with vehicle (10% DMSO in corn oil, n = 8) and Mdivi-1(40 mg/kg, n = 7). Data presented as mean + /−SEM. P value was calculated by Mann–Whitney test. **d** and **e**. Bar graph showing ability of HCC1954 and SKBR3 Pa, Lat, and M-BM to form oncospheres in the presence of HER2 TKIs (lapatinib (2 μM) and tucatinib (3 μM)) and DRP1 inhibitor Mdivi-1 (5 μM) alone or in combination. ‘n’ of individual group has been provided in source data file. ‘n’ in **a** and **c** represents number of mice however in **d** and **e** it represents biologically independent samples. Dotted line indicates control. Data presented as mean + /−SEM. P values were calculated by One-way ANOVA by comparing all groups to control.

Next, we assessed the effect of Mdivi-1 on brain metastasis. To perform this analysis, we injected HCC1954 Lat and M-BM cells intracardially into athymic mice. One week after injection, we administrated Mdivi-1 orally every day for 4 weeks. No significant differences in mice body weights were observed during the course of treatment (Extended Data Fig. 10a). IHC and bioluminescence imaging analyses showed that DRP1 inhibition results in a significant reduction of the number of surviving latent cells and attenuated brain metastatic incidences in mice bearing M-BM cells (Fig. 6d–f and Extended Data Fig. 10b,c). Thus, limiting DRP1 function and consequently impeding mitochondrial plasticity attenuates survival of latent and metachronous brain metastatic cells.

As depletion of DRP1 in Lat and M-BM cells resulted in reduced oncospheres formation and reduced brain metastatic potential of Lat and M-BM cells, we also assessed the impact of the DRP1 inhibitor alone and in combination with the current standard of care^44,45^. HCC1954 and SKBR3 Lat and M-BM cells were resistant to the HER2 tyrosine kinase inhibitors (TKIs) lapatinib or tucatinib as single agents compared to Pa cells. In contrast, Mdivi-1 was effective in limiting oncosphere formation in HCC1954 and SKBR3 Lat and M-BM cells alone and in combination with HER2 TKIs (Extended Data Fig. 10d,e).

**Fig. 7 |:**
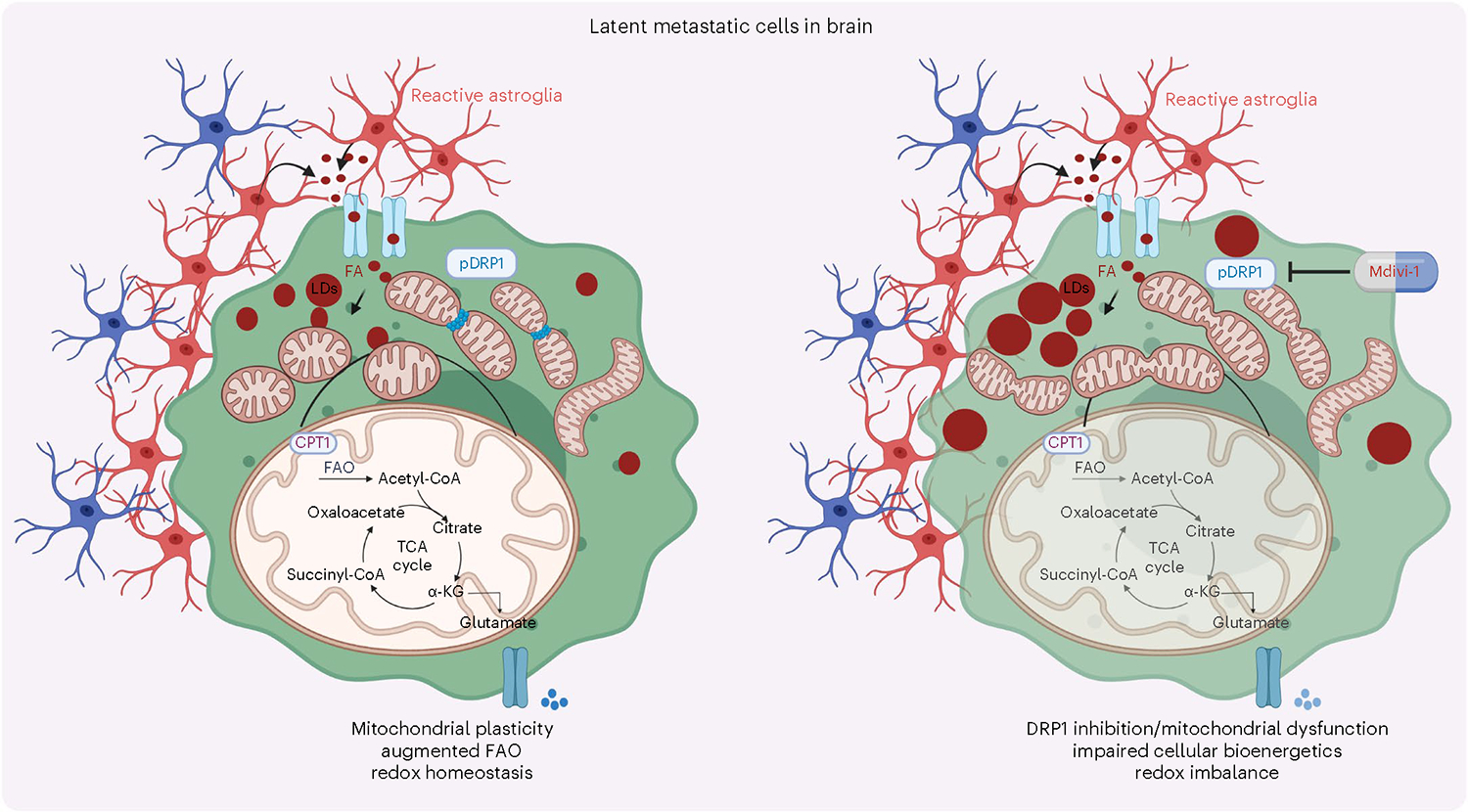
Schematic presentation highlighting the role of DRP1-driven mitochondrial plasticity and metabolic reprogramming in HER2^+^ breast cancer brain metastasis. Illustration was made using BioRender.com.

In summary, our study highlights the vital role of mitochondrial plasticity in facilitating the oxidation of FAs in latent metastatic cells. Depleting or pharmacologically inhibiting DRP1 in preclinical breast cancer metastasis models disrupts mitochondrial dynamics, cellular bioenergetics and redox homeostasis, resulting in attenuated brain metastasis (Fig. 7). These findings suggest that targeting mitochondrial plasticity is a promising therapeutic approach to prevent metastatic recurrences.

## Discussion

Brain metastases are lethal, and their treatment remains an unmet clinical need. Current systemic therapies are primarily directed toward inhibiting oncogenomic alterations or reactivating immune surveillance to limit disease progression. Resistance to targeted therapies are widely observed, and durable responses to immunotherapies are not universal. Moreover, although PTs and metastases share common ancestral driver gene mutations, they evolve independently^46–48^. Reversible adaptations that promote survival and organ colonization are critical for establishing metastasis. We report metabolically flexible, latent disseminated tumor cells rely on mitochondria, the cellular powerhouse and a dynamic organelle, to utilize astrocyte secreted FAs. Fragmented mitochondrial puncta facilitate FAO that supports cellular bioenergetic energy needs and redox homeostasis that is critical for survival of disseminated latent metastatic cells. In agreement with these findings, brain tumor-initiating cells have also been reported to be enriched for fragmented mitochondria, and targeting DRP1 results in increased cell death and reduced tumorigenesis. Moreover, DRP1 activation correlates with poor prognosis in glioblastoma and breast cancers^7,8,49^. These observations highlight targeting mitochondrial dynamics is a viable therapeutic opportunity to limit both brain tumors and metastasis.

Slow-cycling or quiescent latent metastatic cells are also functionally plastic, as they retain ability to proliferate, initiate metastasis and colonize distal organs. Therefore, latent metastasis-promoting alterations are also likely to be reversible and not necessarily driven by fixed oncogenic alterations or signaling responses. Thus, mitochondrial plasticity enables FAO and metabolic programming in response to cellular stress, nutrient state and energy needs and thereby facilitating cellular adaptation is critical for survival of latent disseminated tumor cells. In addition, a consequence of increased FAO is increased pools cellular acetyl-CoA that could reversibly alter signaling responses and epigenomic landscape in metastatic cells^50^.

Overcoming technical limitations to accurately characterize the metabolic state of disseminated latent cells in both mice and humans remain a significant challenge. In this study, we used in vitro metabolic tracing studies to gain a better understanding of the metabolic state of latent cells. However, to further advance our understanding, longitudinal experimental assays to assess the metabolic state of latent and overt metastatic cells are needed. Such assays would provide valuable insights into the metabolic flexibility of disseminated cancer cells and help in developing targeted therapeutic strategies. Likewise, increased DRP1 acetylation is also implicated in promoting mitochondria fission by promoting DRP1 phosphorylation and translocation^33^. Here, we identified several acetylation sites on DRP1 in brain tropic cells. How these modifications affect DRP1 function and the role of mitochondrial dynamics in mediating epigenomic alterations and metastatic latency needs further investigation.

Abnormal mitochondrial dynamics are associated with neurodegenerative diseases, aging and cancer^51–53^. Increased mitochondrial fission in neurons results in mitochondrial dysfunction, and limiting mitochondrial fission results in improved synaptic function and reduced cognitive decline in preclinical models^54^. Moreover, inhibition of mitochondrial fission can also protect myocardial ischemia^33,55,56^ and acute kidney ischemia^57,58^. On the other hand, disseminated Lat breast cancer cells and glioma-initiating cells display fragmented mitochondrial morphology, and DRP1 inhibition results in increased tumor cell death^49^. Thus, targeting DRP1 and mitochondrial fission may have broader therapeutic applicability to cancers with a propensity to disseminate and colonize brain. We show pharmacological inhibition of mitochondrial plasticity using DRP1 inhibitor Mdivi-1 limits residual disease and delays metastatic relapses in preclinical models. Likewise, high-potency small-molecule DRP1 GTPase inhibitors (Drpitor1, Drpitor1a) could also be effective in limiting brain metastasis^59^. Finally, these findings may also have broader therapeutic application in other cancer types with a tendency to metastasize to the brain.

## Methods

### Ethics statement

The University of Texas Southwestern Medical Center Institutional Animal Care and Use Committee approved this study (Animal Protocol no. 2017–102099). All animal studies were conducted in compliance with the ethical guidelines. The maximal tumor size of animal experiments was 2,000 mm^3^, and all experiments did not exceed this limit. Patients consented to use of any deidentified tumor tissues for research purposes under Vanderbilt Ingram Cancer Center institutional review board-approved protocol (Vanderbilt Ingram Cancer Center no. 160606). Validation studies were performed on deidentified tumor tissues (Supplementary Table 6). Patients were not recruited for this specific study. Ages of the patients were undisclosed.

### Cell lines

HCC1954 (Pa; ATCC, Cat. CRL-2338, Lat, M-BM) and SKBR3 (Pa; ATCC, Cat. HBT-30, Lat, M-BM) cells were cultured in RPMI-1640 (Cat. R8758, Sigma-Aldrich) media supplemented with 10% FBS, 2 mM glutamine, 100 units/l^−1^ penicillin, 100 mg l^−1^ streptomycin and 1 μg ml^−1^ amphotericin B. Cells were maintained in 37 °C incubator with 5% CO_2_ and split every 3 days at 1:4 dilution^6^. Lenti-X-HEK 293 T (ATCC, Cat. CRL-1573) cells were grown in DMEM, high-glucose media supplemented with 1 mM sodium pyruvate, 100 U l^−1^ penicillin, 100 mg l^−1^ streptomycin and 10% FBS. Astrocytes were cultured in poly-l-lysine-coated plates with DMEM high-glucose media, (Cat. D6429, Sigma-Aldrich) supplemented with 10% heat-inactivated FBS (Cat. 10437028, Thermo Fisher Scientific), 100 U l^−1^ penicillin, 100 mg l^−1^ streptomycin (Cat. P0781, Sigma-Aldrich) and astrocyte growth supplement (Cat. 1852, ScienCell Research Laboratories).

### Animals

Female athymic mice (Hsd: athymic nude mice-Foxn1nu) aged 4–5 weeks were purchased from Envigo (Cat. 069) and allowed to acclimate to the animal facilities before experimental procedures. The mice were housed in a UT Southwestern animal facility room under a 12/12-h light/dark cycle, at a temperature of 20–26 °C with 30–50% humidity and provided with ad libitum access to a standard Teklad diet (Envigo, Cat. 2916) and water. A dox diet (Envigo, Cat. TD.08541) was given to induce shRNA-mediated specific knockdown or overexpression of the gene of interest in tumor cells. We monitored animal health once daily throughout the experiment timeline. To isolate astrocytes, we obtained 3- to 4-day-old NSG mice (NOD.Cg-Prkdcscid Il2rgtm1Wjl/SzJ; Cat. 005557, Jackson Laboratory) pups from the UTSW animal breeding facility.

### Isolation and culture of mouse cortical astrocytes

NSG mice pup brains aged 3 or 4 days were isolated and kept in chilled 1× HBSS buffer. Using a stereomicroscope, meninges were peeled off from the cortex, and the cortex hemispheres were extracted, cut into small pieces and dissociated using 1:1 HBSS:trypsin (0.125% final) for 6–8 min at 37 °C with occasional shaking. The cells were centrifuged at 400 *g* for 5 min and then grown in poly-l-lysine-coated T75 flasks using astrocyte culture media. The media was changed daily for the first 3 days to remove cellular debris.

### BODIPY-558/568-C12 transfer assays

Astrocytes were cultured for 48 h in six-well plates with complete media, followed by treatment with 2 μM BODIPY-558/568 (Cat. D3835, Thermo Fisher Scientific) for 24 h. After washing the plates three times in warm media, the cells were incubated for 1 h in R3F (RPMI 3% FBS, 10 mM glucose and 0.2 mM glutamine) media. Cancer cells, cultured in R3F for 48 h, were detached by trypsinization and labeled with green cell tracker dye (Cat. C7025, Thermo Fisher Scientific) and co-cultured with astrocytes in R3F media. Live cell time-lapse imaging was performed using a Nikon CSU-W1 Spinning Disk Confocal at 37 °C and 5% CO_2_.

### BODIPY-558/568-C12 pulse-chase assays

Cancer cells were cultured in R3F (six-well plates) media for 24 h, media was discarded and replenished with fresh R3F media (phenol red-free) and then treated with 1 μM BODIPY-558/568 (Cat. D3835, Thermo Fisher Scientific). Time-lapse imaging was performed using an IncuCyte3 imager for 24 or 48 h.

### Transmission electron microscopy

Briefly, 2 × 10^5^ HCC1954 Pa and Lat cells were cultured in MatTek dishes for 24 h with R3F media followed by treatment of sodium palmitate (100 μM) for 24 h. Cells were fixed on MatTek dishes with 2.5% (v/v) glutaraldehyde in 100 mM sodium cacodylate buffer. Samples were processed following the method described previously^60^. To acquire the images, we used a Tecnai G2 Spirit TEM (FEI) with a LaB6 source operating at 120 kV, using a Gatan Ultrascan CCD camera.

### IF imaging for mitochondria visualization

To visualize mitochondria, 2 × 10^5^ cells were cultured on coverslips and immune-stained with anti-TOMM20 antibody. Cells were fixed in 4% paraformaldehyde, permeabilized with 0.1% Triton-X in PBS, blocked with 3% BSA (Cat. A9647, Sigma-Aldrich), and incubated with the primary antibody. DRP1 mitochondrial localization was observed in live cells stained with MitoTracker Deep Red FM (Cat. M22426, Thermo Scientific) before fixation. after primary antibody staining coverslips were washed and incubated with Alexa Fluor conjugated secondary antibody (antibody information is included in Reporting Summary). Coverslips were mounted and visualized with an EVOS fluorescence microscope.

### Steady-state carnitine-conjugated FA analysis

Next, 3 × 10^6^ cells were plated in R3F media (10 cm dish) and grown for 24 h at 37 °C and 5% CO_2_. After aspirating out the media, cells were washed with 1 ml ice-cold 0.9% NaCl solution and then scraped in 1 ml chilled 80% methanol. The cell lysate/methanol mixture was subjected to three freeze/thaw cycles using liquid nitrogen and a 37 °C water bath, vortexed for 1 min and centrifuged at 13,000 *g* for 10 min in a refrigerated centrifuge. The supernatant was collected, dried using a SpeedVac and subjected to liquid chromatography–mass spectrometry (LC–MS) analysis.

### Cell viability assays

To determine cell proliferation in various conditions, MTT (Cat. M5655, Sigma-Aldrich) assay was performed^61^. CellTiter-Glo luminescent cell viability assay (Cat. G9241, Promega) and Trypan blue exclusion assays were performed as per manufacturer’s protocol.

### ROS measurement

Next, 5 × 10^5^ cells were plated in 60-mm plates for 24 h followed by treatment of 100 μM palmitic acid conjugated with BSA for 24 h. Cells were collected and washed with 3% FBS in PBS and incubated with 5 μM CellROX Deep Red reagent (Cat. C10422, Thermo Fisher Scientific) for 30 min. Postincubation cells were washed twice and fixed with 4% paraformaldehyde for 15 min. Next, cells were washed twice and resuspended in PBS with 3% FBS for flow cytometric analysis (FACSCalibur-Becton Dickinson). Analysis was performed using FlowJo v10 software (Supplementary Fig. 2)

### APC Annexin V/PI staining

Briefly, after treatment, 0.5 × 10^6^ cells were washed in ice-cold 1× PBS and resuspended in 100 μl staining buffer and incubated with 5 μl APC Annexin V (Cat. 640932, BioLegend) and 10 μl PI for 15 min at room temperature in a dark place. Flow cytometric analysis was immediately performed using FACSCalibur (Becton Dickinson). Analysis was performed using BD FACSDiva v9.0.1 software (Supplementary Fig. 3).

### Colorimetric caspase-3 activity assay

Colorimetric caspase-3 activity assay was performed using a kit (Cat. ab39401, Abcam). Briefly, cells were grown without palmitate, and cell lysates were prepared using RIPA buffer. An aliquot of 300 μg protein was used for each condition. DTT and DEVD-p-NA substrates were added according to the manufacturer’s protocol. Samples were mixed well and incubated at 37 °C for 90 min, and finally, OD was measured at 405 nm.

### Total FA profiling in tumor cells by GC–MS

Total FA profiles of neutral lipid content was generated by a modified gas chromatography mass spectrometry (GC–MS) method previously described^62^. Samples were analyzed on an Agilent 7890/5975 C by electron capture negative ionization equipped with a DB-5MS column (40 m × 0.180 mm with 0.18 μm film thickness) from Agilent. Hydrogen was used as the carrier gas. FAs were analyzed in selected ion monitoring mode and normalized to internal standards. Data were processed using MassHunter v.B08 software (Agilent). To obtain the neutral lipid content, a three-phase liquid extraction (3PLE) protocol was used^63^.

### BSA-palmitic acid conjugation

For BSA-palmitic acid conjugate preparation, prewarmed RPMI-1640 media (Biological Industries, Cat. SKU: 01–101-1 A) was stirred at 37–40 °C on a heated magnetic stirrer. Next, 10% BSA (Cat. A6003, Sigma-Aldrich) was added to achieve a final BSA concentration of 0.8%. A stock of 10 mM sodium palmitate (Cat. P9767, Sigma-Aldrich) was prepared by dissolving it in isopropanol: water (1:1). Palmitate was added to the RPMI-1640-BSA media while maintaining the final concentration of isopropanol to 0.5% and palmitate to 100 μM. The solution was stirred for 1 h at 37–40 °C, followed filtration. The same protocol was applied for the preparation of BSA and ^13^C_16_ palmitate (Cat. CLM-6059, Cambridge Isotope Laboratories) conjugate.

### In vitro ^13^C_16_ palmitic acid tracing

For in vitro isotope tracing experiments, 1 × 10^6^ cells were grown in 6-cm dishes with 4 ml R3F media for 24 h. The next day, media was discarded and replenished with R3F palmitic acid media (R3F, 100 μM ^13^C_16_ palmitic acid, 0.8% BSA, 0.5% isopropanol) for 24 h. After incubation, media supernatant was collected, and cells were rapidly washed using 400 μl ice-cold 0.9% NaCl and fixed with 300 μl of 80% chilled LC–MS grade methanol in water. Cells were scraped, collected and preserved at −80 °C. Collected samples were subjected to three rounds of freezing/thawing using liquid nitrogen and 37 °C water bath. Supernatant was collected by centrifuging at 13,000 *g* for 10 min and lyophilized using a SpeedVac (Thermo). Lyophilized samples were resuspended in 100 μl 80:20 acetonitrile/water solution (HPLC grade) for LC–MS analysis.

### FAO assay

To assess FAO, SKBR3 Pa and Lat cells were seeded into 96-well plates. Radiolabeled 1-^14^C palmitic acid (1μCi ml^−1^) was given in PBS supplemented with 10 mM HEPES and 1 mM L-carnitine. Nonradiolabeled substrates were added to obtain final concentrations of palmitic acid (100 μM) and glucose (10 mM). CCCP (3 μM) was added for uncoupled substrate oxidation. CO_2_ was captured in an activated UniFilter-96w GF/B microplate with 1 M NaOH (25 μl per well). MicroBeta2 Microplate Counter (PerkinElmer) was used to measure the radioactivity after addition of scintillation liquid (30 μl; MicroScint PS PerkinElmer).

### Nile red and Oil Red O staining for LDs

For Nile red staining, 4% paraformaldehyde fixed cells were washed and stained with 2 μg ml^−1^ Nile red for 10 min. 5 mg ml^−1^ Oil Red O stock solution was prepared by dissolving it in isopropanol. To make working solution, Oil Red O stock solution was added to distilled water in a ratio of 3:2, kept for 10 min and filtered using Whatman filter paper and 0.45-μm syringe filter. Next, Oil Red O working solution was added to the fixed cells for 10 min and rinsed with distilled water. Cells were washed three times, and DAPI staining was done to visualize nucleus.

### Oncosphere assays

To generate oncospheres, 50–100 cells were resuspended in HuMEC serum-free media supplemented with bFGF (10 ng ml^−1^), EGF (20 ng ml^−1^), insulin (5 μg ml^−1^) and 1xB27 supplement. The suspension was incubated at 37 °C and 5% CO_2_ for 7–8 days. The oncospheres were imaged using EVOS fluorescence microscope. Percentage oncosphere formation was determined either by quantification or CellTiter-Glo assay.

### Western blotting

Cells cultured in R3F for 48 h were lysed with RIPA lysis and extraction buffer added with protease and phosphatase inhibitor. For immunoblotting, 20–35 μg protein was resolved in 7.5–12% SDS-PAGE, transferred onto nitrocellulose membrane (Millipore), using a semi-dry transfer apparatus and blocked in 5% BSA or TBST-milk for 1–2 h. Membranes were washed incubated with primary antibody for overnight at 4 °C on a shaker. Membranes were washed with TBST (three or four times for 10 min), and secondary antibody was added. Next, washing was performed and membranes were developed using West Femto Super Signal (Thermo Fisher Scientific) and a Bio-Rad imager. Images were exported with Image Lab v6.1 software. Detail information about antibodies used for western blotting is included in Reporting Summary.

### Steady-state glutathione measurement

The level of total glutathione reduced (GSH) and oxidized glutathione (GSSG) was measured using a kit (Cat. K264, BioVision) according to the manufacturer’s protocol. All data presented were normalized to 10^6^ cells.

### Paraffinized brain tissue section staining

Paraffinized brain tissue section staining was performed as described before^6^. To prepare the samples for IHC analysis, 5- to 10-μm sections were cut and mounted on slides. The slides were then incubated at 65 °C for 30 min, followed by deparaffinization and rehydration through sequential incubations in xylene (two times for 3 min), 100% ethanol (two times for 3 min), 95% ethanol (two times for 3 min), 70% ethanol (two times for 3 min), 50% ethanol (two times for 3 min) and water (two times for 3 min).Antigen retrieval was carried out by placing the slides in 700 ml antigen retrieval buffer (10 mM Tris HCl, 1 mM EDTA, 10% glycerol, pH 9.0) at 95 °C for 25 min. The slides were then cooled to room temperature for 30 min and rinsed with PBS (two times for 2 min). To block nonspecific binding, the slides were treated with 10% horse serum in 0.1% PBS-T for 1 h at room temperature. The slides were incubated with Anti-GFAP at a dilution of 1:500 in 2% serum PBS-T overnight at 4 °C. The next day, the slides were washed in PBS (three times for 10 min) and incubated with secondary antibody for 1 h at room temperature. Slides were washed with PBS (two times for 10 min) and mounted following Hoechst 33342(Cat. 62249, Thermo Fisher Scientific) staining.

IHC analysis of patient samples was performed using a Dako Auto-stainer Link 48 system. The slides were baked at 60 °C for 20 min, followed by deparaffinization and hydration. Antigen retrieval was performed at pH 9.0 for 20 min using the Dako PT Link. The tissue was then incubated with a peroxidase block and anti-phospho-DRP1^S616^ antibody (Cat. 4494, Cell Signaling Technology) at a dilution of 1:200 for 35 min. Finally, the staining was visualized using the EVOS microscope.

### Mass spectrometry for DRP1 posttranslational modifications

Experiment was performed as described previously^64^. Briefly, DRP1 pull down protein samples from Lat cells were digested overnight with trypsin (Pierce) after reduction and alkylation with DTT and iodoacetamide (Sigma-Aldrich). The resulting samples were subjected to solid-phase extraction cleanup with an Oasis HLB plate (Waters) and then injected onto an Orbitrap Fusion Lumos mass spectrometer coupled to an Ultimate 3000 RSLC-Nano liquid chromatography system. The samples were injected onto a 75 μm inner-diameter, 75-cm-long EasySpray column (Thermo Fisher Scientific) and eluted with a gradient from 1% to 28% buffer (80% (v/v) ACN, 10% (v/v) trifluoroethanol and 0.1% formic acid in water) over 90 min. The positive ion mode was used in operating the mass spectrometer, using a source voltage of 1.8 kV and maintaining an ion transfer tube temperature of 275 °C. The Orbitrap was used to acquire MS scans at a resolution of 120,000. Peptide identification was performed using Proteome Discoverer v2.4 SP1 (Thermo Fisher Scientific), with Sequest HT searching against the human protein database from UniProt (downloaded April 8, 2022; 20,361 entries) with a false-discovery rate cutoff of 1% for all peptides. Phosphorylation of serine, threonine, tyrosine and acetylation of lysine were set as variable modifications.

### Virus production and infection for knockdown and rescue

To generate shRNA knockdown cells, Lenti-X 293 T cells were prepared and co-transfected with either pTRIPZ or pTRIPZ-shRNA of target gene CPT1A (Clone Id: V3THS_359757, V3THS_359760, Horizon Discovery), DRP1 (Clone Id: V2THS_29084, only commercially available and validated construct in Horizon Discovery with high efficiency) with packaging plasmid PAX (7 μg) and envelop plasmid MD2.G (2.7 μg) by lipofection method using lipofectamine 3000 (Invitrogen). Eighteen hours after lipofection, media was replenished with fresh media. Virus particles were harvested after 48-h incubation and precipitated using PEG-it virus precipitation solution (Cat. LV810A-1, System Biosciences). Viral precipitates were resuspended with 100 μl Iscove’s modified Dulbecco’s medium (312440053, Thermo Fisher Scientific). Cancer cells cultured overnight were transduced with 20 μl virus solution in 2 ml growth media with 5 μg ml^−1^ polybrene. After 6-h incubation, cells were replenished with 2–3 ml fresh growth media and incubated for 24 h. Knockdown was induced by 1 μg ml^−1^ dox hydrochloride (Cat. 3072, Sigma-Aldrich) supplementation.

For DRP1 rescue in DRP1-depleted cells, synthetic *Homo sapiens* dynamin 1-like (*DNM1L*), transcript variant 5 (the shRNA targeting site was codon optimized without changing the amino acids to minimize the shRNA binding; Supplementary Table 5) was procured from Integrated DNA Technology and cloned into pINDUCER21 (plasmid 46948) where DRP1 was in frame with HA tag. Virus was produced and infected as described above.

### Intracardiac injections and in vivo mice studies

This experiment was performed as described before^6,23,24^. Briefly, 5 × 10^4^ cells were resuspended in 100 μl 1× PBS were intracardially injected into the left ventricle of mice with the help of 26 G tuberculin syringe. Weekly monitoring of tumor growth and the occurrence of metastasis was measured through bioluminescence imaging. For dox-inducible gene depletion experiments, female athymic mice (Hsd: athymic nude mice-Foxn1nu; Envigo) aged 5–6 weeks were injected intracardially either with TRIPZ control or shRNA or rescue cells. Following a week of injection, mice were fed with dox diet and monitored metastatic incidence using noninvasive small animal imager AMI-HTX. Data were analyzed by aura spectral instrument imaging software (v. 4.0).

### NK depletion experiment

Five- to six-week-old female athymic mice (Hsd: athymic nude mice-Foxn1nu; Envigo) were injected with 5 × 10^4^ Ctrl or DRP1-depleted Lat cells intracardially. Postinjection mice were randomized into groups based on bioluminescence imaging signal. NK depletion was performed 1 day after injection by administrating 100 μl anti-asialo GM1 (FUJIFILM Wako Shibayagi, Cat. 986–10001, RRID:AB_516844) intraperitoneally.

### Orthotopic tumor xenograft model

Briefly, 2×10^7^ cells Ctrl and DRP1-depleted Lat and M-BM cells were resuspended in PBS and Matrigel (1:1 ratio) in 1 ml. Female athymic mice (Hsd: Athymic nude mice-Foxn1nu; Envigo) aged 5–6 weeks were anaesthetized by controlled isoflurane administration through a nose cone in a sterile hood. An incision was made between the fourth and fifth nipple of the mouse to expose the mammary fat pad, and 100 μl cell suspension was injected using a 28 G insulin syringe. Four weeks after injection, tumors were collected and tumor volume and weight was measured.

### Mdivi-1 oral gavage

A total of 5 × 10^4^ cancer cells were intracardially injected into 5- to 6-week-old female athymic mice (Hsd: Athymic nude mice-Foxn1nu; Envigo). Postinjection mice were randomized into two groups based on bioluminescence imaging signal. Mice were then administrated with vehicle control (0.2 ml of 10% DMSO in corn oil) and Mdivi-1 (Cat. S7162, Selleckchem, 40 mg kg^−1^) through oral gavage once daily for 4 weeks.

### Statistics and reproducibility

No statistical method was used to pre-determine sample size, but our sample sizes are similar to those reported in previous publications. Data collection and analysis were not performed blind to the conditions of the experiments and outcome assessment. For in vivo experiments in necessary conditions, mice were randomly grouped. In vitro experiments were not randomized. Individual data points were represented as dots in graphs. All statistical analysis tests used in this study are indicated in the figure legends. Statistical significance between two comparative groups was determined using unpaired *t*-test or Mann–Whitney *U*-test. For statistical comparisons between multiple study groups, ordinary one-way analysis of variance (ANOVA) or Kruskal–Wallis test was used. GraphPad prism 9 software was used for all statistical analysis. No data were excluded from the analysis. Data distribution was assumed to be normal, but this was not formally tested.

## Extended Data

## Supplementary Material

Supplementary figures

Supplementary Table 3

Supplementary Table 2

Supplementary Table 1

Supplementary Table 5

Supplementary Table 4

Supplementary Table 6

upplementary Video 2

Supplementary Video 1

Legends for upplementary Videos

upplementary Video 3

upplementary Video 4

## Data Availability

The RNA-seq data that support the findings of this study have been deposited in the Gene Expression Omnibus under the accession number GSE180098.The mass spectrometry data have been deposited in Mass Spectrometry Interactive Virtual Environment (MassIVE) with accession number MSV000091574. Source data are provided with this paper. All other data supporting findings of this study can be available from the corresponding author on reasonable request.

## References

[1] Massague J & Ganesh K Metastasis-initiating cells and ecosystems. Cancer Discov. 11, 971–994 (2021). 33811127 10.1158/2159-8290.CD-21-0010 PMC8030695

[2] Kim K, Marquez-Palencia M & Malladi S Metastatic latency, a veiled threat. Front. Immunol. 10, 1836 (2019). 31447846 10.3389/fimmu.2019.01836 PMC6691038

[3] Faubert B Metabolic reprogramming and cancer progression. Science 368, eaaw5473 (2020). 32273439 10.1126/science.aaw5473 PMC7227780

[4] Ciminera AK, Jandial R & Termini J Metabolic advantages and vulnerabilities in brain metastases. Clin. Exp. Metastasis 34, 401–410 (2017). 29063238 10.1007/s10585-017-9864-8 PMC5712254

[5] Schild T, Low V, Blenis J & Gomes AP Unique metabolic adaptations dictate distal organ-specific metastatic colonization. Cancer Cell 33, 347–354 (2018). 29533780 10.1016/j.ccell.2018.02.001 PMC5889305

[6] Parida PK Metabolic diversity within breast cancer brain-tropic cells determines metastatic fitness. Cell Metab. 34, 90–105 (2022). 34986341 10.1016/j.cmet.2021.12.001 PMC9307073

[7] Ferraro GB Fatty acid synthesis is required for breast cancer brain metastasis. Nat. Cancer 2, 414–428 (2021). 34179825 10.1038/s43018-021-00183-y PMC8223728

[8] Jin X A metastasis map of human cancer cell lines. Nature 588, 331–336 (2020). 33299191 10.1038/s41586-020-2969-2 PMC8439149

[9] Garcia-Bermudez J, Williams RT, Guarecuco R & Birsoy K Targeting extracellular nutrient dependencies of cancer cells. Mol. Metab. 33, 67–82 (2020). 31926876 10.1016/j.molmet.2019.11.011 PMC7056928

[10] Lehuede C, Dupuy F, Rabinovitch R, Jones RG & Siegel PM Metabolic plasticity as a determinant of tumor growth and metastasis. Cancer Res. 76, 5201–5208 (2016). 27587539 10.1158/0008-5472.CAN-16-0266

[11] Mosier JA, Schwager SC, Boyajian DA & Reinhart-King CA Cancer cell metabolic plasticity in migration and metastasis. Clin. Exp. Metastasis 38, 343–359 (2021). 34076787 10.1007/s10585-021-10102-1

[12] Tilokani L, Nagashima S, Paupe V & Prudent J Mitochondrial dynamics: overview of molecular mechanisms. Essays Biochem. 62, 341–360 (2018). 30030364 10.1042/EBC20170104 PMC6056715

[13] Altieri DC Mitochondrial dynamics and metastasis. Cell. Mol. Life Sci. 76, 827–835 (2019). 30415375 10.1007/s00018-018-2961-2 PMC6559795

[14] Porporato PE, Filigheddu N, Pedro JMB, Kroemer G & Galluzzi L Mitochondrial metabolism and cancer. Cell Res. 28, 265–280 (2018). 29219147 10.1038/cr.2017.155 PMC5835768

[15] Brosnan EM & Anders CK Understanding patterns of brain metastasis in breast cancer and designing rational therapeutic strategies. Ann. Transl. Med. 6, 163 (2018). 29911111 10.21037/atm.2018.04.35 PMC5985267

[16] Zimmer AS HER2-positive breast cancer brain metastasis: A new and exciting landscape.Cancer Rep. 5, e1274 (2020). 10.1002/cnr2.1274 PMC9124511 32881421

[17] Kuksis M The incidence of brain metastases among patients with metastatic breast cancer: a systematic review and meta-analysis. Neuro. Oncol. 23, 894–904 (2021). 33367836 10.1093/neuonc/noaa285 PMC8168821

[18] Ho VK Survival of breast cancer patients with synchronous or metachronous central nervous system metastases. Eur. J. Cancer 51, 2508–2516 (2015). 26277099 10.1016/j.ejca.2015.07.040

[19] Kodack DP The brain microenvironment mediates resistance in luminal breast cancer to PI3K inhibition through HER3 activation. Sci. Transl. Med. 9, eaal4682 (2017). 28539475 10.1126/scitranslmed.aal4682 PMC5917603

[20] Lin NU, Gaspar LE & Soffietti R Breast cancer in the central nervous system: Multidisciplinary considerations and management. Am. Soc. Clin. Oncol. Educ. Book 37, 45–56 (2017). 28561683 10.1200/EDBK_175338

[21] Olson E & Mullins DA When standard therapy fails in breast cancer: Current and future options for HER2-positive disease. J. Clin. Trials 3, 1000129–1000129 (2013). 24527366 10.4172/2167-0870.1000129 PMC3920550

[22] Kabraji S Drug resistance in HER2-positive breast cancer brain metastases: Blame the barrier or the brain. Clin. Cancer Res. 24, 1795–1804 (2018). 29437794 10.1158/1078-0432.CCR-17-3351 PMC5899637

[23] Malladi S Metastatic latency and immune evasion through autocrine inhibition of WNT. Cell 165, 45–60 (2016). 27015306 10.1016/j.cell.2016.02.025 PMC4808520

[24] Parida PK Optimized protocol for stable isotope tracing and steady-state metabolomics in mouse HER2^+^ breast cancer brain metastasis. STAR Protoc. 3, 101345 (2022). 35496802 10.1016/j.xpro.2022.101345 PMC9048131

[25] Er EE Author Correction: Pericyte-like spreading by disseminated cancer cells activates YAP and MRTF for metastatic colonization. Nat. Cell Biol. 21, 408 (2019). 10.1038/s41556-018-0257-2 30542103

[26] Koundouros N & Poulogiannis G Reprogramming of fatty acid metabolism in cancer. Br. J. Cancer 122, 4–22 (2020). 31819192 10.1038/s41416-019-0650-z PMC6964678

[27] Escartin C Reactive astrocyte nomenclature, definitions, and future directions. Nat. Neurosci. 24, 312–325 (2021). 33589835 10.1038/s41593-020-00783-4 PMC8007081

[28] Henrik Heiland D Tumor-associated reactive astrocytes aid the evolution of immunosuppressive environment in glioblastoma. Nat. Commun. 10, 2541 (2019). 31186414 10.1038/s41467-019-10493-6 PMC6559986

[29] Wasilewski D, Priego N, Fustero-Torre C & Valiente M Reactive astrocytes in brain metastasis. Front. Oncol. 7, 298 (2017). 29312881 10.3389/fonc.2017.00298 PMC5732246

[30] Zou Y Polyunsaturated fatty acids from astrocytes activate PPARgamma signaling in cancer cells to promote brain metastasis. Cancer Discov. 9, 1720–1735 (2019). 31578185 10.1158/2159-8290.CD-19-0270 PMC6891206

[31] Olzmann JA & Carvalho P Dynamics and functions of lipid droplets. Nat. Rev. Mol. Cell Biol. 20, 137–155 (2019). 30523332 10.1038/s41580-018-0085-z PMC6746329

[32] Listenberger LL Triglyceride accumulation protects against fatty acid-induced lipotoxicity. Proc. Natl Acad. Sci. USA 100, 3077–3082 (2003). 12629214 10.1073/pnas.0630588100 PMC152249

[33] Hu Q Increased Drp1 acetylation by lipid overload induces cardiomyocyte death and heart dysfunction. Circ. Res. 126, 456–470 (2020). 31896304 10.1161/CIRCRESAHA.119.315252 PMC7035202

[34] Xie L Drp1-dependent remodeling of mitochondrial morphology triggered by EBV-LMP1 increases cisplatin resistance. Signal Transduct. Target. Ther. 5, 56 (2020). 32433544 10.1038/s41392-020-0151-9 PMC7237430

[35] Benador IY Mitochondria bound to lipid droplets have unique bioenergetics, composition, and dynamics that support lipid droplet expansion. Cell Metab 27, 869–885 (2018). 29617645 10.1016/j.cmet.2018.03.003 PMC5969538

[36] Nguyen D A new vicious cycle involving glutamate excitotoxicity, oxidative stress and mitochondrial dynamics. Cell Death Dis. 2, e240 (2011). 22158479 10.1038/cddis.2011.117 PMC3252734

[37] Unger RH, Clark GO, Scherer PE & Orci L Lipid homeostasis, lipotoxicity and the metabolic syndrome. Biochim. Biophys. Acta 1801, 209–214 (2010). 19948243 10.1016/j.bbalip.2009.10.006

[38] He L Carnitine palmitoyltransferase-1b deficiency aggravates pressure overload-induced cardiac hypertrophy caused by lipotoxicity. Circulation 126, 1705–1716 (2012). 22932257 10.1161/CIRCULATIONAHA.111.075978 PMC3484985

[39] Dobbins RL Prolonged inhibition of muscle carnitine palmitoyltransferase-1 promotes intramyocellular lipid accumulation and insulin resistance in rats. Diabetes 50, 123–130 (2001). 11147777 10.2337/diabetes.50.1.123

[40] Rappold PM Drp1 inhibition attenuates neurotoxicity and dopamine release deficits in vivo. Nat. Commun. 5, 5244 (2014). 25370169 10.1038/ncomms6244 PMC4223875

[41] Wang W Parkinson’s disease-associated mutant VPS35 causes mitochondrial dysfunction by recycling DLP1 complexes. Nat. Med. 22, 54–63 (2016). 26618722 10.1038/nm.3983 PMC4826611

[42] Grohm J Inhibition of Drp1 provides neuroprotection in vitro and in vivo. Cell Death Differ. 19, 1446–1458 (2012). 22388349 10.1038/cdd.2012.18 PMC3422469

[43] Macia E Dynasore, a cell-permeable inhibitor of dynamin. Dev. Cell 10, 839–850 (2006). 16740485 10.1016/j.devcel.2006.04.002

[44] Cordero A Combination of tucatinib and neural stem cells secreting anti-HER2 antibody prolongs survival of mice with metastatic brain cancer. Proc. Natl Acad. Sci. USA 119, e2112491119 (2022). 34969858 10.1073/pnas.2112491119 PMC8740706

[45] Kulukian A Preclinical activity of HER2-selective tyrosine kinase inhibitor tucatinib as a single agent or in combination with trastuzumab or docetaxel in solid tumor models. Mol. Cancer Ther. 19, 976–987 (2020). 32241871 10.1158/1535-7163.MCT-19-0873

[46] Reiter JG Minimal functional driver gene heterogeneity among untreated metastases. Science 361, 1033–1037 (2018). 30190408 10.1126/science.aat7171 PMC6329287

[47] Brastianos PK Genomic characterization of brain metastases reveals branched evolution and potential therapeutic targets. Cancer Discov. 5, 1164–1177 (2015). 26410082 10.1158/2159-8290.CD-15-0369 PMC4916970

[48] Nguyen B Genomic characterization of metastatic patterns from prospective clinical sequencing of 25,000 patients. Cell 185, 563–575 e511 (2022). 35120664 10.1016/j.cell.2022.01.003 PMC9147702

[49] Xie Q Mitochondrial control by DRP1 in brain tumor initiating cells. Nat. Neurosci. 18, 501–510 (2015). 25730670 10.1038/nn.3960 PMC4376639

[50] Xiong X Activation of Drp1 promotes fatty acids-induced metabolic reprograming to potentiate Wnt signaling in colon cancer.Cell Death Differ. 29, 1913–1927 (2022). 35332310 10.1038/s41418-022-00974-5 PMC9525627

[51] Chan DC Mitochondria: dynamic organelles in disease, aging, and development. Cell 125, 1241–1252 (2006). 16814712 10.1016/j.cell.2006.06.010

[52] Sebastian D, Palacin M & Zorzano A Mitochondrial dynamics: coupling mitochondrial fitness with healthy aging. Trends Mol. Med. 23, 201–215 (2017). 28188102 10.1016/j.molmed.2017.01.003

[53] Chen H & Chan DC Mitochondrial dynamics in regulating the unique phenotypes of cancer and stem cells. Cell Metab 26, 39–48 (2017). 28648983 10.1016/j.cmet.2017.05.016 PMC5539982

[54] Baek SH Inhibition of Drp1 ameliorates synaptic depression, Aβ deposition, and cognitive impairment in an Alzheimer’s disease model. J. Neurosci. 37, 5099–5110 (2017). 28432138 10.1523/JNEUROSCI.2385-16.2017 PMC6596467

[55] Ong SB Inhibiting mitochondrial fission protects the heart against ischemia/reperfusion injury. Circulation 121, 2012–2022 (2010). 20421521 10.1161/CIRCULATIONAHA.109.906610

[56] Maneechote C, Palee S, Chattipakorn SC & Chattipakorn N Roles of mitochondrial dynamics modulators in cardiac ischaemia/reperfusion injury. J. Cell. Mol. Med. 21, 2643–2653 (2017). 28941171 10.1111/jcmm.13330 PMC5661112

[57] Bhatia D, Capili A & Choi ME Mitochondrial dysfunction in kidney injury, inflammation, and disease: Potential therapeutic approaches. Kidney Res. Clin. Pract. 39, 244–258 (2020). 32868492 10.23876/j.krcp.20.082 PMC7530368

[58] Perry HM Dynamin-related protein 1 deficiency promotes recovery from AKI. J. Am. Soc. Nephrol. 29, 194–206 (2018). 29084809 10.1681/ASN.2017060659 PMC5748924

[59] Wu D Identification of novel dynamin-related protein 1 (Drp1) GTPase inhibitors: Therapeutic potential of Drpitor1 and Drpitor1a in cancer and cardiac ischemia-reperfusion injury. FASEB J. 34, 1447–1464 (2020). 31914641 10.1096/fj.201901467R

[60] Nair VR Microfold cells actively translocate Mycobacterium tuberculosis to initiate infection. Cell Rep. 16, 1253–1258 (2016). 27452467 10.1016/j.celrep.2016.06.080 PMC4972672

[61] Parida PK Inhibition of cancer progression by a novel trans-stilbene derivative through disruption of microtubule dynamics, driving G2/M arrest, and p53-dependent apoptosis. Cell Death Dis. 9, 448 (2018). 29670107 10.1038/s41419-018-0476-2 PMC5906627

[62] Quehenberger O, Armando AM & Dennis EA High sensitivity quantitative lipidomics analysis of fatty acids in biological samples by gas chromatography-mass spectrometry. Biochim. Biophys. Acta 1811, 648–656 (2011). 21787881 10.1016/j.bbalip.2011.07.006 PMC3205314

[63] Vale G Three-phase liquid extraction: a simple and fast method for lipidomic workflows. J. Lipid Res. 60, 694–706 (2019). 30610084 10.1194/jlr.D090795 PMC6399505

[64] Cannavino J Regulation of cold-induced thermogenesis by the RNA binding protein FAM195A. Proc. Natl Acad. Sci. USA 118, e2104650118 (2021). 34088848 10.1073/pnas.2104650118 PMC8201964

