# Supplementary material for: Limiting mitochondrial plasticity by targeting DRP1 induces metabolic reprogramming and reduces breast cancer brain metastases
Source: Nat Cancer. Author manuscript; Available in PMC 2024 Jul 31. (PMC11290463; doi:10.1038/s43018-023-00563-6)
Supplement: Supplementary figures [file NIHMS2007169-supplement-Supplementary_figures.pdf]

Supplementary Fig. 1a

a

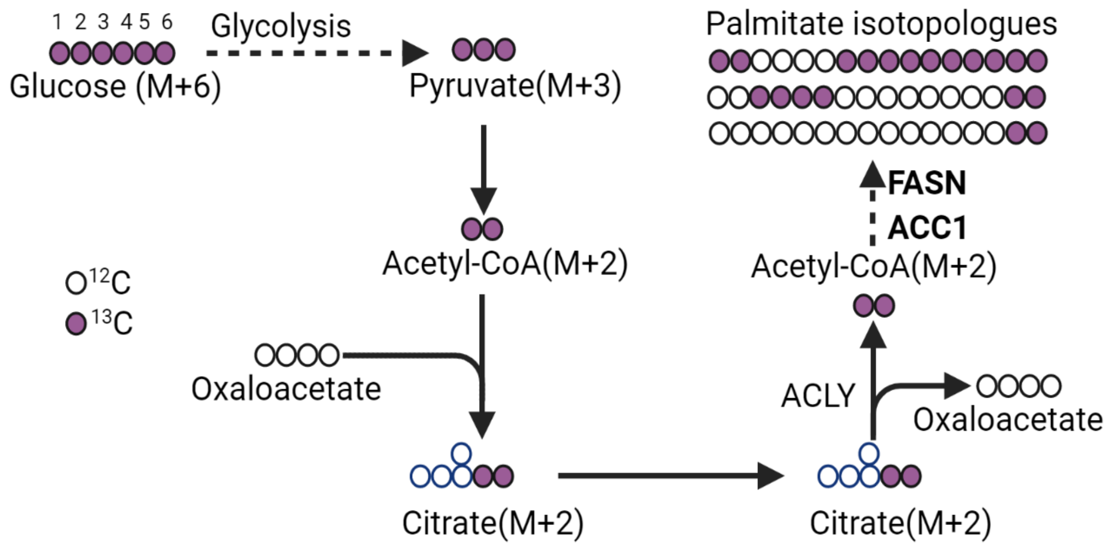

b

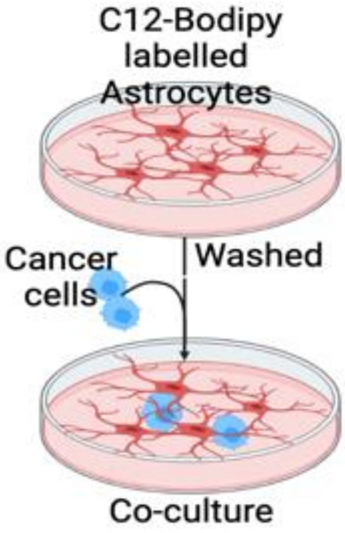

Supplementary Fig. 1: a. Schematic representation showing 13C6-glucose contribution to palmitate isotopologues. Illustration was made using BioRender (BioRender.com) licence. **b.**Schematic representation showing methods used for astrocyte and cancer cell co-culture. Illustration was made using BioRender (BioRender.com) licence.

Supplementary Fig. 2

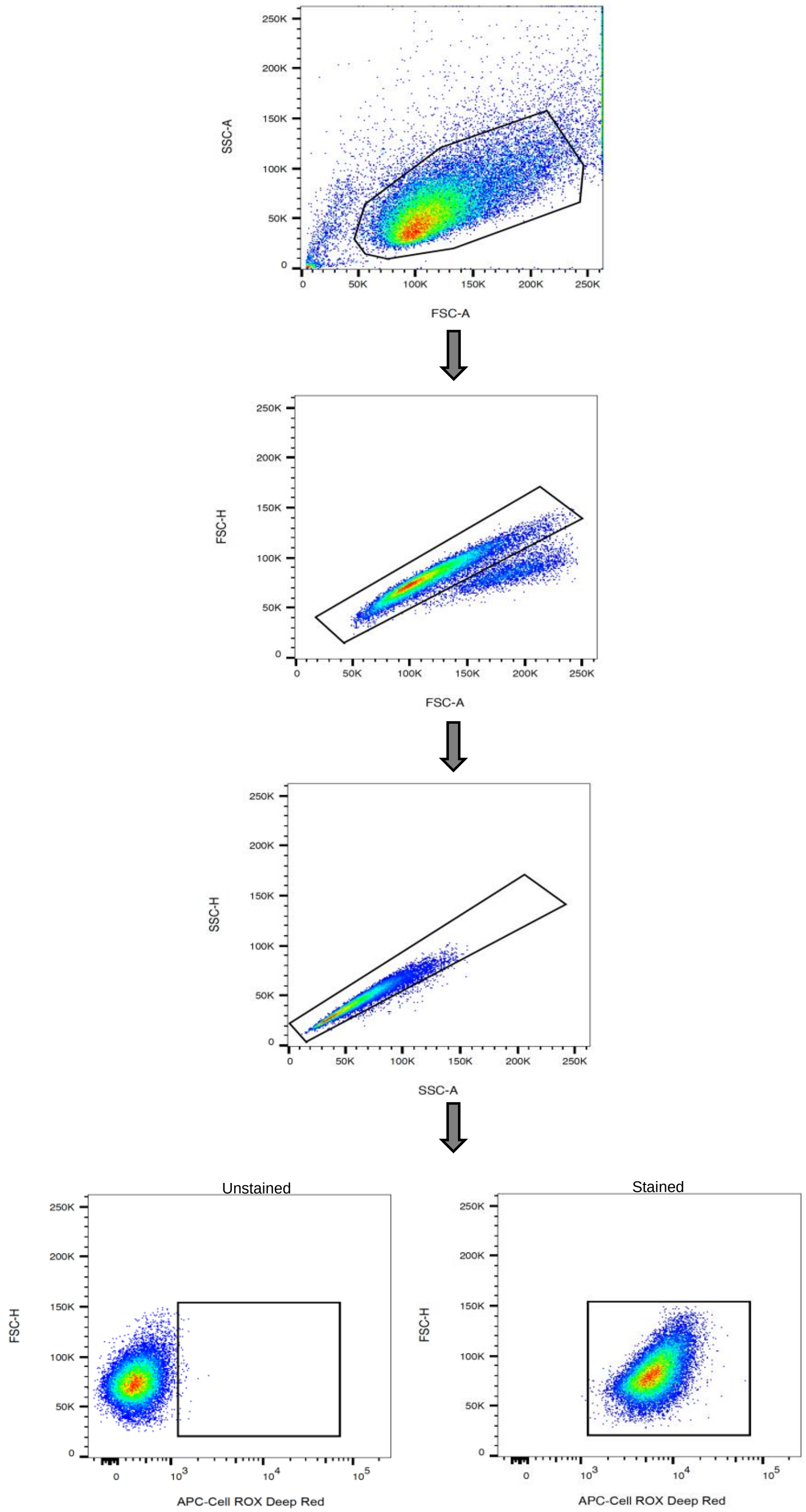

Supplementary Fig. 2: Gating Strategy: Reactive Oxygen Species Measurement. Doublets were excluded by FSC-A versus FSC-H gating. Cell ROX Deep Red positive cells were further analyzed. Unstained cells were considered negative staining control. Analysis was performed in FlowJo v10 software.

Supplementary Fig. 3

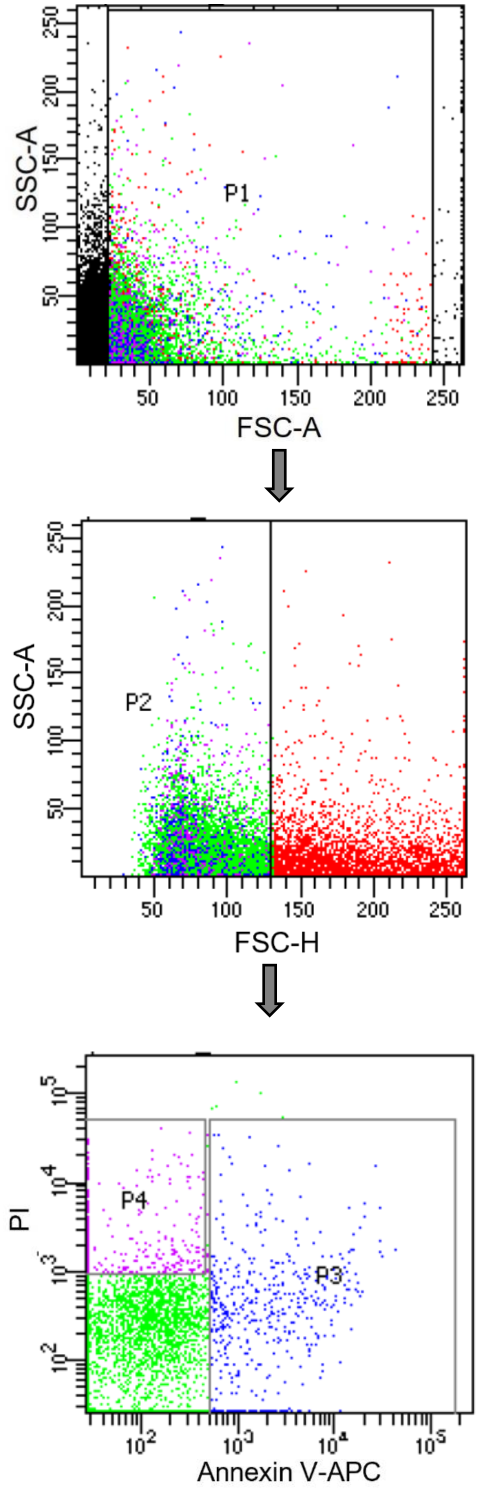

Supplementary Fig. 3: Gating Strategy for APC Annexin V/PI staining. Doublets were excluded by FSC-A versus FSC-H gating. Percentage (%) of Annexin V/PI positive cells were calculated. Analysis was performed in BD FACSDiva v9.0.1 software.
