# Supplementary material for: Limiting mitochondrial plasticity by targeting DRP1 induces metabolic reprogramming and reduces breast cancer brain metastases
Source: Nat Cancer. Author manuscript; Available in PMC 2024 Jul 31. (PMC11290463; doi:10.1038/s43018-023-00563-6)
Supplement: Legends for upplementary Videos [file NIHMS2007169-supplement-Legends_for_upplementary_Videos.docx]

**Legends for Supplementary Videos**

**Supplementary Video (AVI files)**

**Supplementary Video 1a.** Confocal microscope time lapse imaging showing lipid transfer from astrocytes to HCC1954 Pa cells in co-culture setting. (Bright field: Astrocytes, Green: Pa cells, Red: BODIPY-C12).

**Supplementary Video 1b.** Confocal microscope time lapse imaging showing lipid transfer from astrocytes to HCC1954 Lat cells in co-culture setting. (Bright field: Astrocytes, Green: Lat cells, Red: BODIPY-C12).

**Supplementary Video 1c.** IncuCyte3 time lapse images showing lipid uptake and cell death in HCC1954 Pa cells cultured with 2μM of BODIPY-558/568-C12. (Bright field: Pa cells, Red: BODIPY-C12).

**Supplementary Video 1d.** IncuCyte3 time lapse images showing lipid uptake and cell death in HCC1954 Lat cells cultured with 2μM of BODIPY-558/568-C12. (Bright field: Lat cells, Red: BODIPY-C12).
